# The *Toxoplasma gondii* dense granule protein TgGRA3 interacts with host Golgi and dysregulates anterograde transport

**DOI:** 10.1242/bio.039818

**Published:** 2019-02-27

**Authors:** Maika S. Deffieu, Tchilabalo Dilezitoko Alayi, Christian Slomianny, Stanislas Tomavo

**Affiliations:** 1 Center for Infection and Immunity of Lille, CNRS UMR 8204, INSERM U1019, Université de Lille, 59 000 Lille, France; 2 Plateforme de Protéomique et Peptides Modifiés (P3M), CNRS, Université de Lille, 59000 Lille, France; 3 Laboratory of Cell Physiology, INSERM U 1003, Université de Lille, 59655 Villeneuve d'Ascq, France; 4 Institute for Integrative Biology of the Cell (I2BC), CNRS UMR 9198, CEA, Université Paris Sud, Université Paris-Saclay, 91198 Gif-sur-Yvette Cedex, France

**Keywords:** *Toxoplasma gondii*, Dense granules, Secretion, Host Golgi, Anterograde transport

## Abstract

After entry into the host cell, the intracellular parasite *Toxoplasma gondii* resides within a membrane-bound compartment, the parasitophorous vacuole (PV). The PV defines an intracellular, parasite-specific niche surrounded by host organelles, including the Golgi apparatus. The mechanism by which *T. gondii* hijacks the host Golgi and subverts its functions remains unknown. Here, we present evidence that the dense granule protein TgGRA3 interacts with host Golgi, leading to the formation of tubules and the entry of host Golgi material into the PV. Targeted disruption of the Tg*GRA3* gene delays this engulfment of host Golgi. We also demonstrate that TgGRA3 oligomerizes and binds directly to host Golgi membranes. In addition, we show that TgGRA3 dysregulates anterograde transport in the host cell, thereby revealing one of the mechanisms employed by *T. gondii* to recruit host organelles and divert their functions.

This article has an associated First Person interview with the first author of the paper.

## Abstract (precis)

**Summary** : *Toxoplasma gondii* recruits various host organelles to enable parasite intracellular development. We describe a new role for TgGRA3 in modulating the host anterograde transport by binding to the Golgi apparatus.

## INTRODUCTION

Membrane trafficking pathways regulate diverse key cellular functions, including endocytosis, transport of cargo, metabolism and immunity. Intracellular pathogens, including protozoan parasites, have the capacity to subvert these functions during infection of a host cell.

*Toxoplasma gondii*, an obligate intracellular protozoan parasite, is the causative agent of toxoplasmosis, an asymptomatic disease principally dangerous to fetuses of primo-infected mothers and immunocompromised patients such as those infected with HIV. *Toxoplasma gondii* contains specific secretory organelles named rhoptries (ROP), micronemes (MIC) and dense granules (GRA). These organelles are necessary for actively mediated entry of the parasite into mammalian cells through mechanisms that bypass the phagocytic pathway. This entry process creates a membrane-bound compartment called the parasitophorous vacuole (PV). The PV originates from the host plasma membrane and defines a parasite-specific compartment within the infected cell. *Toxoplasma gondii* secretes proteins to reshape the PV membrane (PVM) (Suss-Toby et al., 1996; Charron and Sibley, 2004; Carruthers and Boothroyd, 2007). The PVM does not fuse to the host cell degradative system, thereby avoiding endolysosomal lysis (Sinai and Joiner, 1997; Clough and Frickel, 2017).

During parasite growth, the composition of the PV and its luminal space is further modified through the secretion of proteins and lipids (Sibley, 2011; Clough and Frickel, 2017; Hakimi et al., 2017). In addition, an intravacuolar network (INV) characterized by numerous tubules forms inside the PV (Sibley et al., 1995; Mercier et al., 2002). Secretion of two dense granule proteins, TgGRA2 and TgGRA7, into the PV stabilizes the INV (Mercier et al., 2002; Travier et al., 2008). Lipids retrieved from the host cell by the parasite extensively build up the INV (Caffaro and Boothroyd, 2011). In addition, the presence of parasite-derived protein complexes increases PVM porosity to host cytosol components (Schwab et al., 1994; de Souza and Attias, 2015; Gold et al., 2015). Within a few hours after entry, the parasite begins to recruit various host organelles into the PV (de Melo et al., 1992; Melo and de Souza, 1997; Sinai et al., 1997; Melo et al., 2001).

*Toxoplasma gondii* is auxotrophic for many metabolites, and its survival and growth depend on how successfully it can scavenge nutrients from the host cell (Blader and Koshy, 2014; Coppens, 2014). For example, Coppens et al. (2006) showed that *T. gondii* captures host lysosomes that pass through the PVM and are delivered to the luminal space of the PV, allowing the parasite to scavenge cholesterol from the infected host cell. In addition, *T. gondii* salvages sphingolipids from the host Golgi by rerouting selected Rab vesicles to the PV (Coppens et al., 2000; Romano et al., 2013)*.* Although host cholesterol is required for the intracellular development of the parasite, sphingolipids appear not to be essential, as they are also synthesized *de novo* by *T. gondii* (Mina et al., 2017). As a consequence, inhibition of host sphingolipid synthesis only weakly decreases parasite replication (Romano et al., 2013). Moreover, other host organelles such as mitochondria, the endoplasmic reticulum (ER), endosomes and the Golgi apparatus are recruited within minutes after parasite entry into the host cell (Melo and de Souza, 1997; de Melo et al., 1997; Sinai et al., 1997; Romano et al., 2013). The dense granule protein TgMAF-1 plays a key role in recruiting host mitochondria to the PV (Pernas et al., 2014). Overall, the tight association between host organelles and the PVM facilitates nutrient exchange and membrane fusion to deliver host compounds into the PV, where the parasite replicates (Sinai et al., 1997; Gold et al., 2015). However, it remains to be determined how *T. gondii* recruits host cell organelles and subverts their functions.

In this study, we developed biochemical and proteomic approaches for identifying *T. gondii* proteins that bind host Golgi membranes. Using these techniques, we identified the dense granule protein TgGRA3 and revealed its interaction with host Golgi. In particular, we found that TgGRA3 coats tubular structures inside the PV that contain host Golgi material. We also demonstrated that TgGRA3 causes dysregulation of the host cell's anterograde pathway, which is involved in protein secretion from the ER to the plasma membrane. Thus, our observations provide insight into one of the mechanisms used by the intracellular parasite *T. gondii* to modulate functions of host organelles.

## RESULTS

### Identification of parasite proteins associated with host Golgi membranes

**Fig. 1.**
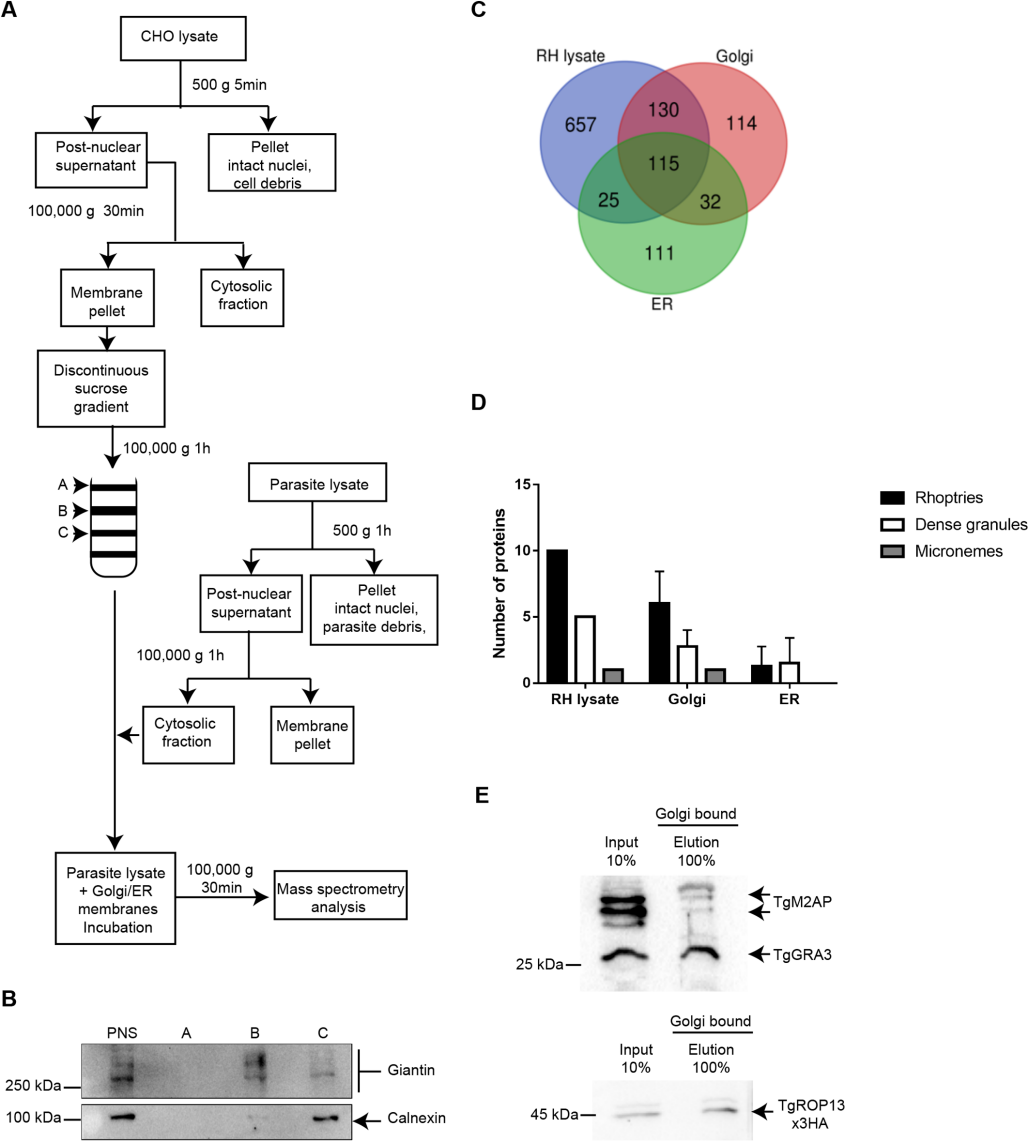
**Identification of parasite proteins that interact with the host Golgi apparatus.** (A) Protocol for identifying parasite proteins that bind host membrane fractions. CHO cells were lysed, and after differential centrifugation, total membranes were layered on a discontinuous sucrose gradient. The Golgi apparatus and endoplasmic reticulum (ER) were collected. In parallel, parasite cytosolic fractions containing parasite proteins were also collected. Fractions B and C from the sucrose gradient, containing the enriched Golgi and ER membranes, respectively, were incubated with parasite lysate, and then pelleted and analyzed by mass spectrometry. (B) Western blot analysis after organelle fractionation. Antibodies specific to calnexin (ER marker) and giantin (cis-Golgi marker) were used. The anti-golgin antibodies recognize multiple glycoprotein forms, whereas the anti-calnexin antibodies bind a single protein. (C) Venn diagram summarizing mass spectrometry data from quadruplicate experiments, representing parasite proteins identified in the input parasite lysate (RH), host Golgi-enriched membranes, and host ER-enriched membranes. (D) Classification of the number of secreted proteins (rhoptry, dense granule and microneme) identified in the mass spectrometry data in the input parasite lysate, Golgi-enriched membranes, and ER-enriched membranes. Results are presented as numbers of proteins±s.d. (E) Identification of parasite proteins binding to host Golgi membranes. Immunoblots were probed with anti-HA (for TgROP13), anti-M2AP, and anti-GRA3 antibodies.

To identify *T. gondii* proteins that interact with the host Golgi apparatus, we incubated Golgi membranes purified from non-infected CHOK-1 cells with parasite extracts. Because *T. gondii* also has the ability to recruit the ER, we harvested non-infected CHOK-1 cells and separated Golgi membranes from the ER membranes by subcellular fractionation (Fig. 1A). Immunoblotting of the collected fractions detected the cis-Golgi marker giantin in fraction B, whereas the ER-resident protein calnexin was mainly present in fraction C (Fig. 1B). Notably in this regard, giantin is a glycoprotein that appears as multiple glycosylated forms, leading to multiple stained bands migrating above 250 kDa, whereas calnexin migrates as a single band of 100 kDa (Fig. 1B). Weak ER contamination was present in fraction B (Fig. 1B). Host Golgi-enriched (fraction B) or ER-enriched (fraction C) samples were incubated with protein extracted from *T. gondii* parasites (strain RH). After several washing steps to remove non-specifically bound protein, membrane samples from the binding assay, as well as the parasite RH lysate alone were subjected to proteomic analysis by nanoscale liquid chromatography coupled to tandem mass spectrometry (nanoLC-MS/MS). Direct analysis of RH lysate alone identified 927 proteins: 657 proteins present in RH lysate alone; 130 proteins present in RH lysate alone and Golgi-bound proteins; 115 proteins present in RH lysate alone, Golgi-bound proteins, and ER-bound proteins; and 25 proteins present in RH lysate alone and ER-bound proteins (Fig. 1C; Table S1). These data were generated from quadruplicate assays followed by mass spectrometry. We concluded that 130 *T. gondii* proteins could potentially bind to CHOK-1 Golgi-enriched membranes, whereas only 25 *T. gondii* proteins could bind to CHOK-1 ER-enriched membranes. Thirty-two *T. gondii* proteins that commonly bound to both Golgi-enriched membranes and ER-enriched membrane were not present in RH lysate alone (Fig. 1C; Table S1); accordingly, these latter proteins were not investigated further.

To identify proteins of special interest, we applied the following criteria: (1) selected proteins must have been identified in RH lysate and in CHOK-1 Golgi-enriched membrane (Golgi)-binding assays at least twice in four independent experiments; (2) they must have been identified at most twice in CHOK-1-enriched ER (ER)-binding assays; and (3) proteins that were identified twice in both CHOK-1 Golgi-enriched membrane (Golgi)- and CHOK-1-enriched ER (ER)-binding assays were excluded. Using these criteria, we limited the list to 49 selected proteins (Table S2). Classification of the secreted proteins according to their previously known localization revealed that several GRA, ROP, and MIC proteins attached to host Golgi-enriched membranes (Fig. 1D).

Among these putative host Golgi-interacting protein candidates, RON proteins (TgRON5, TgRON8, TgRON2), and the microneme protein MIC1 are involved in entry of the parasite into the host cell (Saouros et al., 2005; Straub et al., 2011; Poukchanski et al., 2013; Beck et al., 2014). The Golgi-enriched fraction also bound to dense granule proteins TgGRA3 and TgROP18, which are localized at the PV. Previous work showed that TgROP18 is a kinase that targets immunity-related GTPases (IRGs) (Hermanns et al., 2016), whereas the function of TgGRA3, a putative single-transmembrane protein, was unknown. Knockout of Tg*GRA3* attenuates virulence in mice but causes no obvious phenotype *in vitro* (Craver and Knoll, 2007). TgGRA3 was also identified in a yeast two-hybrid screen as a potential ER-interacting protein (Kim et al., 2008). TgGRA3 is secreted into the intravacuolar network, and is localized at the PVM and on PVM projections in the host cytosol (Dunn et al., 2008). Based on these observations, TgGRA3 was a promising candidate for host organelle interaction.

In addition, we chose to further investigate the role of a second protein, TgROP13, even though it was not identified as a candidate by our criteria after mass spectrometry. Specifically, it was detected only once in the Golgi-binding assay. However, previous work identified TgROP13 as a secreted protein of unknown function localized at the PVM and secreted into the host cell (Turetzky et al., 2010). Therefore, in our investigation of the function of TgGRA3 in recruiting of host Golgi, TgROP13 was used as a control (Turetzky et al., 2010). To confirm that host Golgi membranes could bind to TgGRA3 and TgROP13, we carried out the binding assay described above. To visualize TgROP13, we chromosomally appended a 3×HA epitope tag to the gene. This knock-in strategy enables expression of epitope-tagged protein under the control of the native promoter at a level comparable to that of the endogenous protein. Next, we incubated Golgi membranes with detergent-free parasite lysates prepared from wild-type (RH) parasites and the TgROP13-3×HA transgenic parasites. Immunoblotting confirmed that TgGRA3 and TgROP13-3×HA bound host Golgi-enriched membranes (Fig. 1E). As a negative control, the microneme protein TgM2AP, which was not identified among the mass spectrometry candidates, bound only weakly to these Golgi-enriched membranes (Fig. 1E). However, we also observed binding of TgGRA3 to ER-enriched membranes, as previously reported by Kim et al. (2008). We have not further investigated this aspect of ER-binding. Instead, we focus our investigation on the significance of host Golgi-binding by TgGRA3.

### TgGRA3 localizes in filamentous projections directed towards host Golgi

**Fig. 2.**
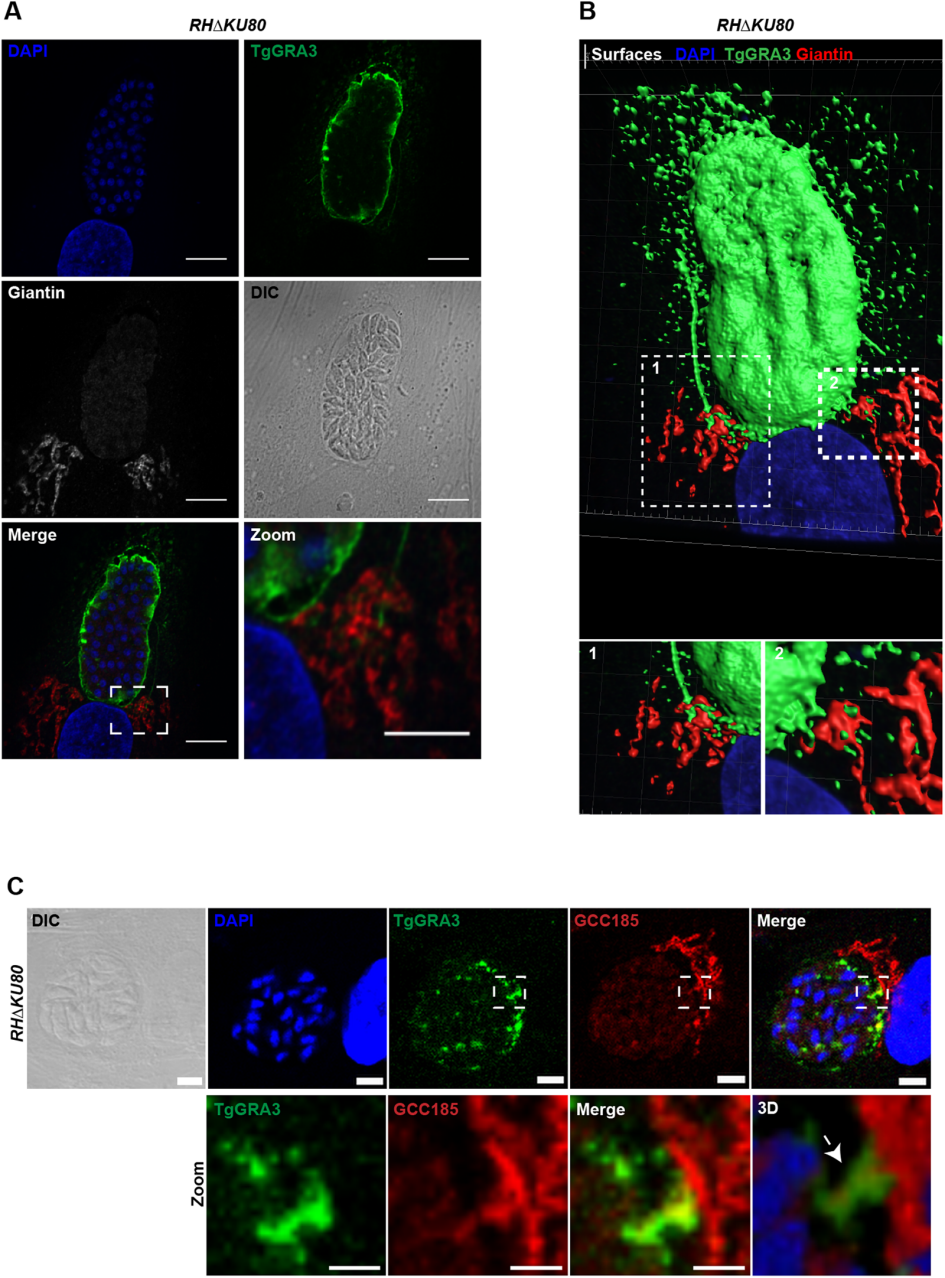
**TgGRA3 is present in PV projections and PVM tubules.** (A) Z-stack confocal images of HFF cells infected with the RHΔ*KU80* parental strain for 30 h. The host Golgi marker giantin (gray) and TgGRA3 (green) were visualized using specific antibodies, and nuclei were stained with DAPI. The white dashed square corresponds to a magnified region. The gamma value was changed to 0.75 in the magnified image to better visualize the TgGRA3-labeled structures. Scale bars: 10 µm (2 µm for magnified regions). (B) Surfaces of A created after three-dimensional reconstruction, showing TgGRA3 (green) at the PVM and in PV projections. White dashed squares represent magnified regions 1 and 2, which depict PV projections and PV filaments, respectively, containing TgGRA3 in contact with the host Golgi. (C) Z-stack confocal image of HFF cells infected with the RHΔ*KU80* parental strain for 35 h before fluorescence experiments, as above. Cells were fixed and permeabilized with 0.1% Triton X-100 at 37°C to visualize the INV. The host Golgi marker GCC185 (red) and TgGRA3 (green) were visualized using specific antibodies, and nuclei were stained with DAPI. The white dashed square represents a magnified region (lower panel) showing TgGRA3 co-localizing with GCC185. Scale bars: 2 µm. Three-dimensional reconstruction of the magnified region indicating TgGRA3 coating a tubule (white arrow) in the INV, which contains host Golgi marker GCCC185.

We compared the localizations of TgGRA3 and TgROP13 to that of the Golgi marker giantin using confocal microscopy (Fig. 2; Fig. S1A). TgGRA3 was detected at the PVM along with filamentous projections towards the host Golgi apparatus (Fig. 2A, dashed squares indicate magnified area). TgROP13 was also localized to the PVM (Fig. S1B), but no obvious interactions between the host Golgi and TgROP13 were observed like those seen in TgGRA3 staining (Fig. 2B, dashed squares indicate magnified area). These filamentous projections of the PVM towards the host Golgi, though of unknown function, have previously been described (Dubremetz et al., 1993). We noticed that a small amount of TgGRA3 staining was associated with other uncharacterized vesicular structures originating around the PV (Fig. 2B). These structures seemed to be in close contact with host Golgi membranes (Fig. 2B). By using confocal microscopy, we also observed co-localization between TgGRA3 and golgin GCC185 (Fig. 2C, magnified square). After three-dimensional reconstruction from optical z-slices, this co-localized region formed a TgGRA3-coated tubule surrounding the host Golgi material at the PVM (Fig. 2C, white arrow; Fig. S2, also see Movies 1 and 2).

### Loss of *GRA3* impairs host Golgi recruitment and entry

**Fig. 3.**
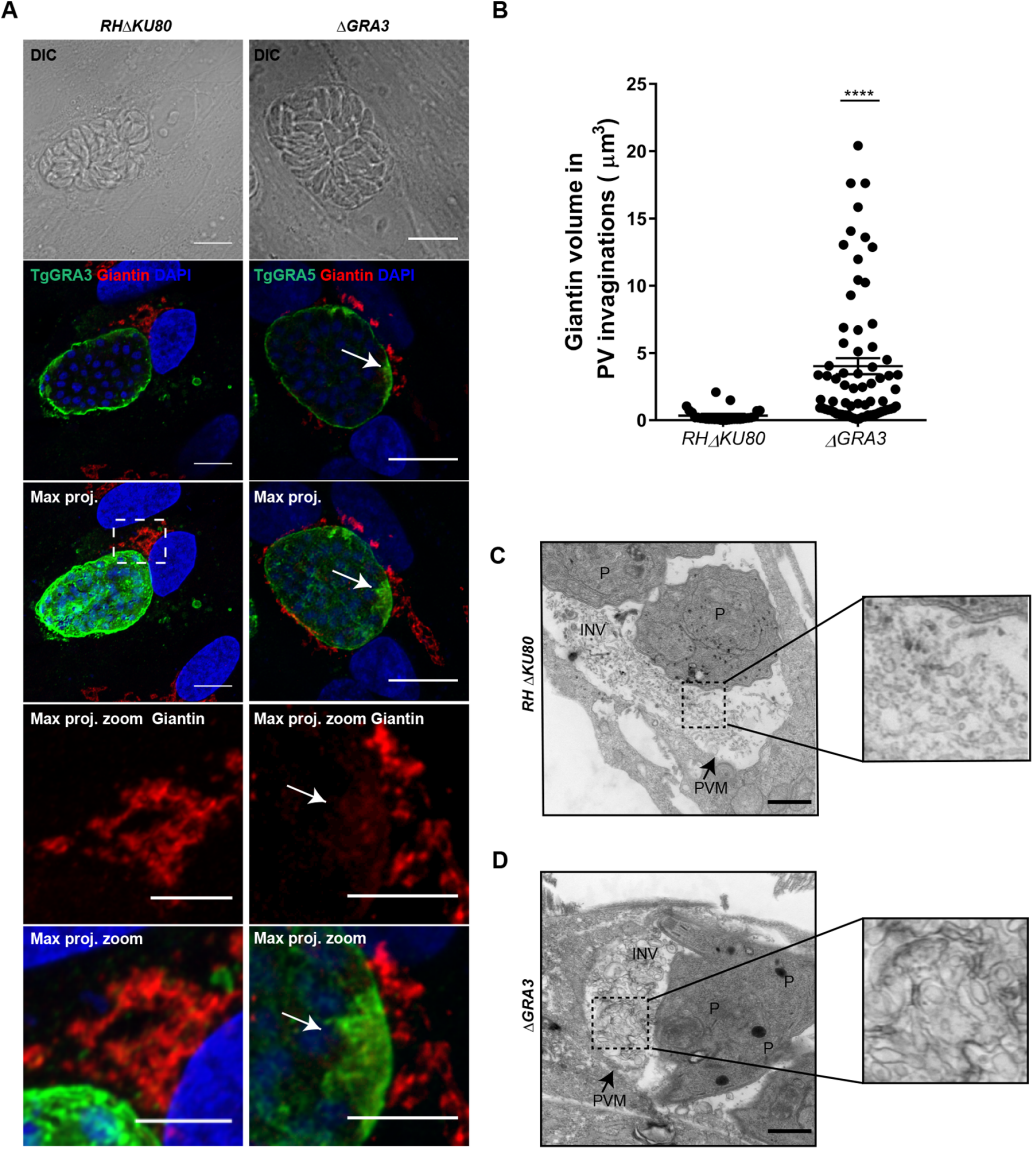
**TgGRA3 depletion accumulates host Golgi material at the PVM.** (A) A representative z-stack of deconvolved confocal images of HFF cells infected with RHΔ*ku80* parental strain and Δ*GRA3* parasites for 30 h. The host Golgi marker giantin (red), PV marker TgGRA5 (green), and nuclei (DAPI) were stained. ‘Max. proj.’ indicates maximal projections of all z-stacks. White dashed squares indicate magnified regions showing TgGRA3 proximity to the host Golgi for the parental strain, and giantin-positive invagination at the PVM (white arrow) for Δ*GRA3* parasites. Scale bars: 10 µm (5 µm in magnified regions). (B) Histograms quantifying PV invagination volume for RHΔ*KU80* parental strain and Δ*GRA3* parasites 30 h post-infection. Results are presented as percentages±s.e.m. 20 parasitophorous vacuoles from three independent experiments were measured. ****P<0.0001. (C) Transmission electron micrograph showing parasitophorous vacuole containing intracellularly growing *RH*Δ*KU80* parental parasites. Black dashed square indicates intravacuolar tubules from RHΔ*KU80* parental parasites. The magnified image of this region is presented in the right panel. (D) Transmission electron micrograph showing that TgGRA3 depletion in Δ*GRA3* mutant accumulates vesicles at the INV. Black dashed square indicates large vesicular structures from Δ*GRA3* mutants. The magnified image of this region is presented in the right panel. P, parasites; PVM, parasitophorous vacuole membrane; INV, intravacuolar network. Scale bars: 500 nm.

To determine the role of Tg*GRA3* gene in host Golgi recruitment, we replaced endogenous Tg*GRA3* with the dihydrofolate reductase resistance cassette (*DHFR*) (Fig. S1C). Disruption of the *TgGRA3* gene was verified by PCR (Fig. S1D) and the absence of protein expression was confirmed in these Δ*GRA3* mutants by confocal microscopy (Fig. S1E, lower panel) compared to the parental parasites, which are still expressing the protein (Fig. S1E, upper panel), as expected. Host cell Golgi localization was analyzed in cells infected with these Δ*GRA3* mutants. Because TgGRA5 has a localization similar to that of TgGRA3 (Fig. S1F), we used TgGRA5 as a PVM marker in Δ*GRA3* mutants. We noticed a higher accumulation of host Golgi material around the vacuole containing Δ*GRA3* mutants (Fig. 3A, right panels, white arrow). In contrast, host Golgi was compactly clustered between the host nucleus and the vacuole occupied by the parental parasites expressing TgGRA3 protein normally (Fig. 3A, left panels). Hence, we observed giantin-positive invaginations in the vacuoles containing Δ*GRA3* mutants. Quantification of these giantin-labeled invaginations revealed that they were almost fourfold larger in volume (4.025 µm^3^±0.60) than those in parental parasites (0.553 µm^3^±0.08) (Fig. 3B). Because we observed that TgGRA3 coats tubules originating from the PVM, we hypothesized that accumulation of host Golgi material at the PVM in Δ*GRA3* mutants may be due to a defect in the formation of these tubules. Ultrastructural microscopy revealed that intracellular Δ*GRA3* mutants contained large vesicles and exhibited abnormal morphology (Fig. 3D) relative to intracellular parental parasites (Fig. 3D), which had normal PV tubular networks (dashed square, magnified square). The data suggest that TgGRA3 may be required for formation of normal tubules at the PVM.

### TgGRA3 conditional depletion affects PV entry of host Golgi vesicles

**Fig. 4.**
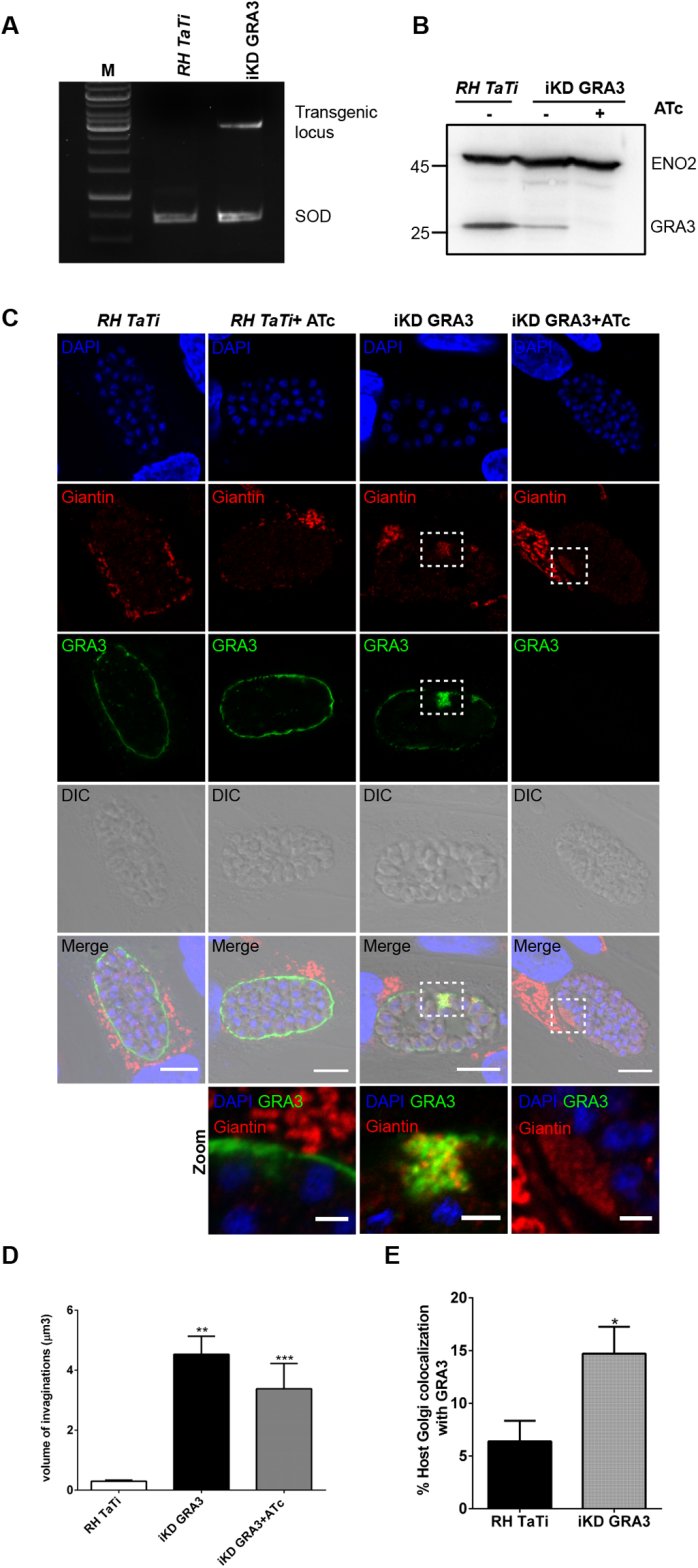
**Inducible TgGRA3 depletion affects host Golgi recruitment and entry at the PVM.** (A) Agarose gel of PCR showing correct integration of the transgene into Tg*GRA3* locus. SOD (superoxide dismutase) is used as a PCR loading control. (B) Western blot analysis demonstrating the decrease in TgGRA3 protein level in iKD-GRA3 mutants. ATc treatment depleted TgGRA3 protein expression in iKD-GRA3 mutants. Eno2 is used as a loading control. (C) Representative z-stack from confocal microscopy images of HFF cells infected with *RH TaTi* parental strain and iKD-GRA3 in absence or in presence of ATc. At 35 h post-infection, iKD-GRA3 parasites indicated abnormal accumulations of host Golgi material at the PVM (white dashed squares). A magnification of this region is presented in the lower panel. The host Golgi marker giantin (red), PV marker TgGRA5 (green), and nuclei (DAPI) were stained. Scale bars: 10 µm (5 µm in magnified regions). (D) Histogram quantifying PV invagination volume. Invagination volumes (*n*=80) were measured from three-dimensional reconstructions of 30 parasitophorous vacuoles for each condition, **P value<0.0001, ***P value=0.0005 (unpaired *t*-test). Results are reported±s.e.m. (E) Histogram quantifying the percentage of co-localization between TgGRA3 and the cis-Golgi marker giantin. Three-dimensional reconstructions were done on 12 parasitophorous vacuoles for each condition, *P value=0.0242 (unpaired *t*-test). Results are reported±s.e.m.

We also decided to use another strategy to generate an inducible time-dependent TgGRA3 knockdown mutants (designated iKD-GRA3) that will reduce protein expression. In this conditional knockdown mutants, Tg*GRA3* gene expression is driven by the tetO7SAG4 promoter sensitive to anhydrotetracycline (ATc) (Fig. 4A; Fig. S3A). Because the tetO7SAG4 promoter is weaker than the endogenous TgGRA3 promoter, this replacement of promoter significantly decreased the level of TgGRA3 protein compared to the parental strain without ATc addition (Fig. 4B). The level of ENO2 protein was used as loading protein control (Fig. 4B). As expected, the addition of ATc completely abolished TgGRA3 protein expression. To explore the consequences of the conditional TgGRA3 knockdown on host Golgi recruitment, we visualized host Golgi localizations relative to the PVM in cells infected by the iKD-GRA3 mutants (Fig. 4C). During the host Golgi recruitment process, we observed different phenotypes of host Golgi localization that we classified as ‘far’, ‘top’ or ‘around’. In the ‘far’ phenotype the host Golgi is not recruited at the PV, while at the ‘top’ phenotype the host Golgi is located at one side of the vacuole. The ‘around’ phenotype describes the host Golgi surrounding the PV (Fig. 4C, upper panels). We observed that at 35 h post-infection most of the host Golgi was recruited. We noticed that most of the host Golgi was located to the PV in the absence (76.75%±8.13) and presence of ATc (79.00%±22.62) in control parasites. In untreated intracellular iKD-GRA3 mutants, 57.50%±31.80 of Golgi membranes were around the PV compared to 38.50%±16.26 around the PV in ATc treated conditions (Fig. 4C, see histogram in bottom panel). Significantly, 47.65%±11.81 of the ATc-treated iKD-GRA3-infected cells displayed host Golgi with a PV ‘top’ phenotype relative to 8.65%±6.15 of cells infected with ATc-treated parental parasites (Fig. 4C, bottom merged and magnified panels). Collectively, these data on host Golgi recruitment observed in iKD-TgGRA3 mutants are in good agreement with those obtained in Δ*GRA3* mutants (Fig. 3, also see Fig. 7H) where the ‘far’, ‘top’ and ‘around’ phenotypes were more explicitly described. Clearly, we observed the presence of accumulated host Golgi material at the PVM in the iKD-GRA3 mutants (Fig. 4C, white dashed squares). In contrast, the parental strain (*RH* TaTi) contained small giantin-positive vesicles, which had a total volume of 0.29 µm^3^±0.03 while in the iKD-GRA3 mutant, the volume of accumulated giantin at the PVM was 15-fold higher and measured at 4.50 µm^3^±0.60 (Fig. 4D). Treatment of the iKD-GRA3 mutants with ATc (3.37 µm^3^±0.84) did not significantly change the volume of giantin-positive structures from the untreated condition. Thus, the accumulation of giantin at the PVM suggests that the decrease in TgGRA3 level affects the entry of host Golgi vesicles. Moreover, we noticed that the accumulated giantin principally localized to PVM regions with concentrated TgGRA3. Consequently, in the iKD-GRA3, we observed an increase in the extent of co-localization between TgGRA3 and giantin (14.71%±2.54) when compared to the parental *RH* TaTi control (6.38%±1.96) (Fig. 4E).

### TgGRA3 forms a homodimer through its coiled-coil domain

**Fig. 5.**
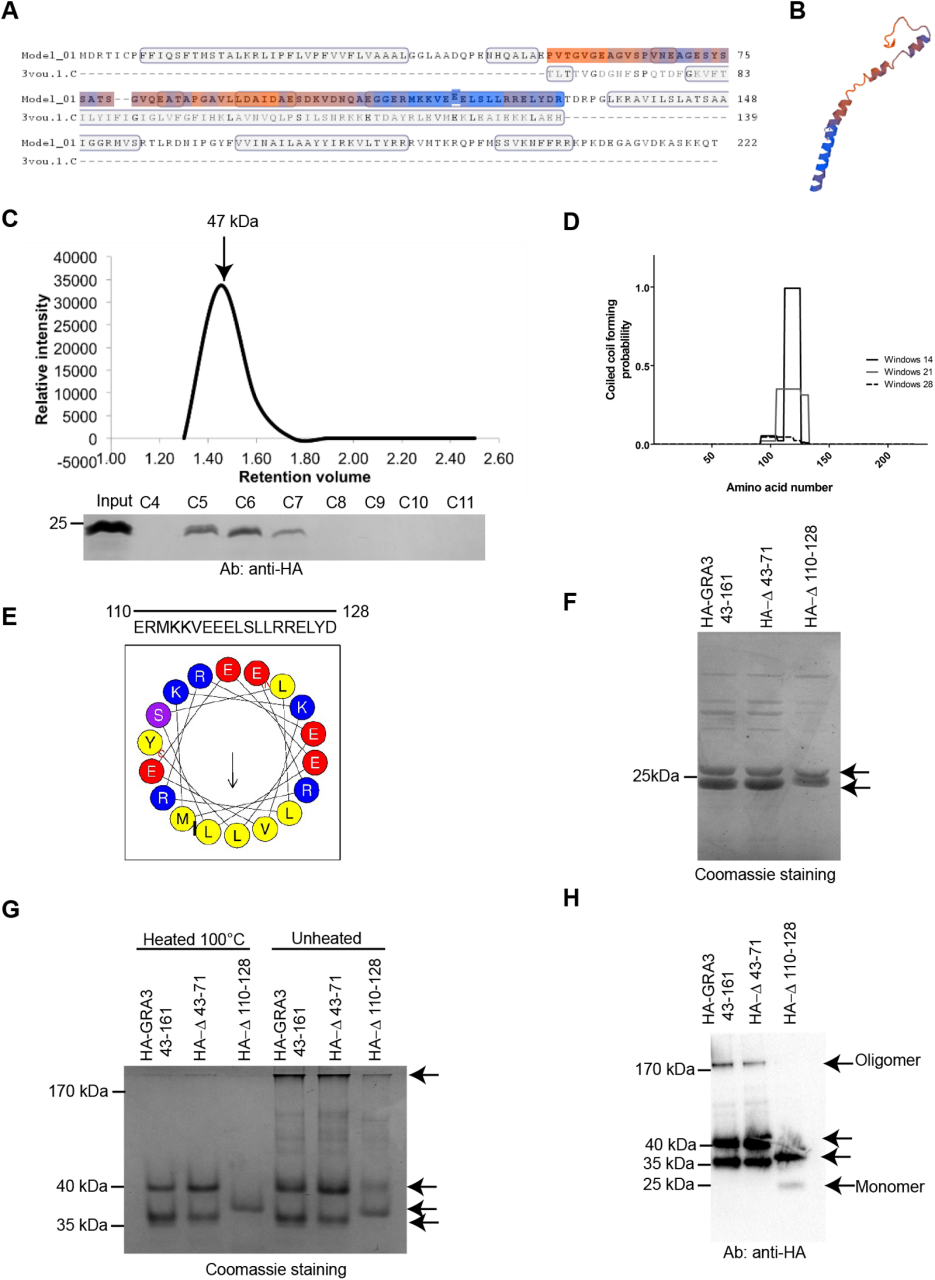
**The C-terminal domain of TgGRA3 is necessary for protein oligomerization.** (A) Protein modeling of TgGRA3 identified a region similar (colored region) to the C-terminal region of the voltage-gated sodium channel NavSulP. (B) A putative structure of this region in A was established according to structural homology with NavSulP. (C) Size-exclusion chromatography of GRA3_43-161_. GRA3_43-161_ eluted as a single peak of 47 kDa, suggesting dimerization of the protein in solution. (D) COILS software prediction of coiled-coil regions in the TgGRA3 protein. (E) Helical wheel depicting the putative coiled-coil region of TgGRA3. Yellow, hydrophobic regions; blue, positively charged amino acids; red, negatively charged amino acids; purple, polar amino acids. (F) Denaturing gel showing purified HA-GRA3_43-161_ proteins and two mutated versions, HA- Δ110-128 and HA-Δ43-71. All proteins were present as doublets (black arrows). (G) Native PAGE analyzing oligomerization state of purified proteins (HA-GRA3_43-161_, HA-Δ43-71, and HA-Δ110-128). Proteins were denatured by heating and kept in the native state under unheated conditions. The gel was stained with Coomassie Blue. Black arrows indicate doublet forms observed in the denaturing gel. (H) Western blots of native PAGE gels showing the oligomerization states HA-GRA3_43-161_, HA-Δ110-128, and HA-Δ43-71. Black arrows indicate various protein forms that were observed. The oligomeric form disappeared in the absence of the coiled-coil region.

To have further insights on TgGRA3 function, we analyzed the putative structure of the TgGRA3 protein using HHpred and SWISS-MODEL. We found a coiled-coil region (colored region) that was structurally similar (18.67% identity) to the C-terminal region of the voltage-gated sodium channel (PDB structure 3VOU.1c) (Fig. 5A). A putative structure of this region of TgGRA3 was established according to structural homology with NavSulP (Fig. 5B), a voltage-gate sodium channel whose oligomeric state is stabilized by coiled-coil regions (Irie et al., 2012). Notably, TgGRA3 can form oligomers at the PVM (Ossorio et al., 1994). However, the domain of TgGRA3 responsible for its oligomerization has not yet been identified. Therefore, we generated a TgGRA3 construct lacking the signal sequence and single-transmembrane domain (GRA3_43-161_) (Fig. S4A) and fused to GST for the protein purification. After purification, the recombinant protein did not migrate at the predicted molecular weight of 39 kDa under denaturing conditions consistent with another report (Yang et al., 2014) (Fig. S4B,C). The GST-cleaved form of GRA3_46-161_ that was predicted to be 12 kDa migrated as a doublet at 24 kDa (Fig. S4B). To achieve complete denaturation, we pre-treated the protein with DTT or 8 M urea, but observed no change in the molecular weight of the protein (Fig. S4D), suggesting that the protein's mobility under denaturing conditions was influenced by its biochemical properties.

We then carried out gel filtration to analyze the oligomeric state of GRA3_46-161_ cleaved from GST (Fig. 5C). The protein was eluted as a single peak between fractions C5 and C7, corresponding to a molecular weight of 47 kDa, suggesting that the protein might exist in a dimeric state in solution.

Coiled-coil regions often mediate protein oligomerization (Mason and Arndt, 2004). Analysis of the primary sequence of TgGRA3 using the COILS algorithm (Lupas et al., 1991) revealed a putative coiled-coil region between amino acids 110 and 128 (Fig. 5D). Analysis using HeliQuest (Keller, 2015) indicated that this region contains a highly hydrophobic sequence consisting of the amino acids MLLVL (Fig. 5E). To identify TgGRA3 oligomerization sites, we compared the behavior of HA-GRA3_43-161_ protein with those of the N-terminally truncated GRA3_HA-Δ43-71 (HA-Δ43-71) and the C-terminal deletion GRA3_HA-Δ110-128 (HA-Δ110-128), which lacks the coiled-coil region. All three proteins behaved as a doublet on denaturing gels (Fig. 5F). We also analyzed the oligomeric state of these proteins using native PAGE. Both HA-GRA3_43-161_ and HA-Δ43-71 migrated as doublets at 35 and 40 kDa. We hypothesized that these doublets could be two different structural forms of the protein having different mobilities in the native gel electrophoresis. Only the band at 35 kDa remained after deletion of the coiled-coil region (HA-Δ110-128), suggesting a role of the coiled coil region in this other structural form (Fig. 5G). Under native conditions (unheated), a lower amount of a high-molecular weight band corresponding to an oligomeric form appeared above 180 kDa only for HA-GRA3_43-161_ and HA-Δ43-71 (Fig. 5H), whereas the HA-Δ110-128 mutant migrated as a 35 kDa band and a low amount of a 24 kDa band that corresponds to the monomeric form (Fig. 5H).

To determine the effect of the expression of these mutants *in vivo*, we complemented ΔGRA3 parasites with a GRA3-HA wild-type gene as well as the GRA3-Δ110-128- HA mutants (denominated comp-Δ110-128- HA) (Fig. S5). The parasites showed a correct localization of GRA3-HA and GRA3-Δ110-128 proteins at the PVM (Fig. S5A, ortho-view). However, we noticed that the expression of GRA3-HA and GRA3-Δ110-128 was not homogeneous compare to the parental strain as we noticed concentrated regions at the PVM (Fig. S5B, white arrows). We also notice that the host Golgi was still accumulating at the PVM in the GRA3-HA wild type and GRA3-Δ110-128 mutants (Fig. S5B, Movie 3). When we quantified the volume of these invaginations, we found a significant increase in the volume of host Golgi material incorporated in the vacuoles containing the complemented with the GRA3-HA (10.84 µm^3^±2.15) mutants compared to the one observed in the *ΔGRA3* mutant alone (4.02 µm^3^±0.60). However, the GRA3-Δ110-128 (4.93 µm^3^±0.87) showed no significant difference compare to the ΔGRA3 (Fig. S5C). Altogether, these results indicate that localization of TgGRA3 at the PVM could modulate host Golgi entry at the PVM as these accumulations appeared to be at TgGRA3 concentrated regions.

### TgGRA3 binds to phosphatidylinositol lipids through its C-terminus

**Fig. 6.**
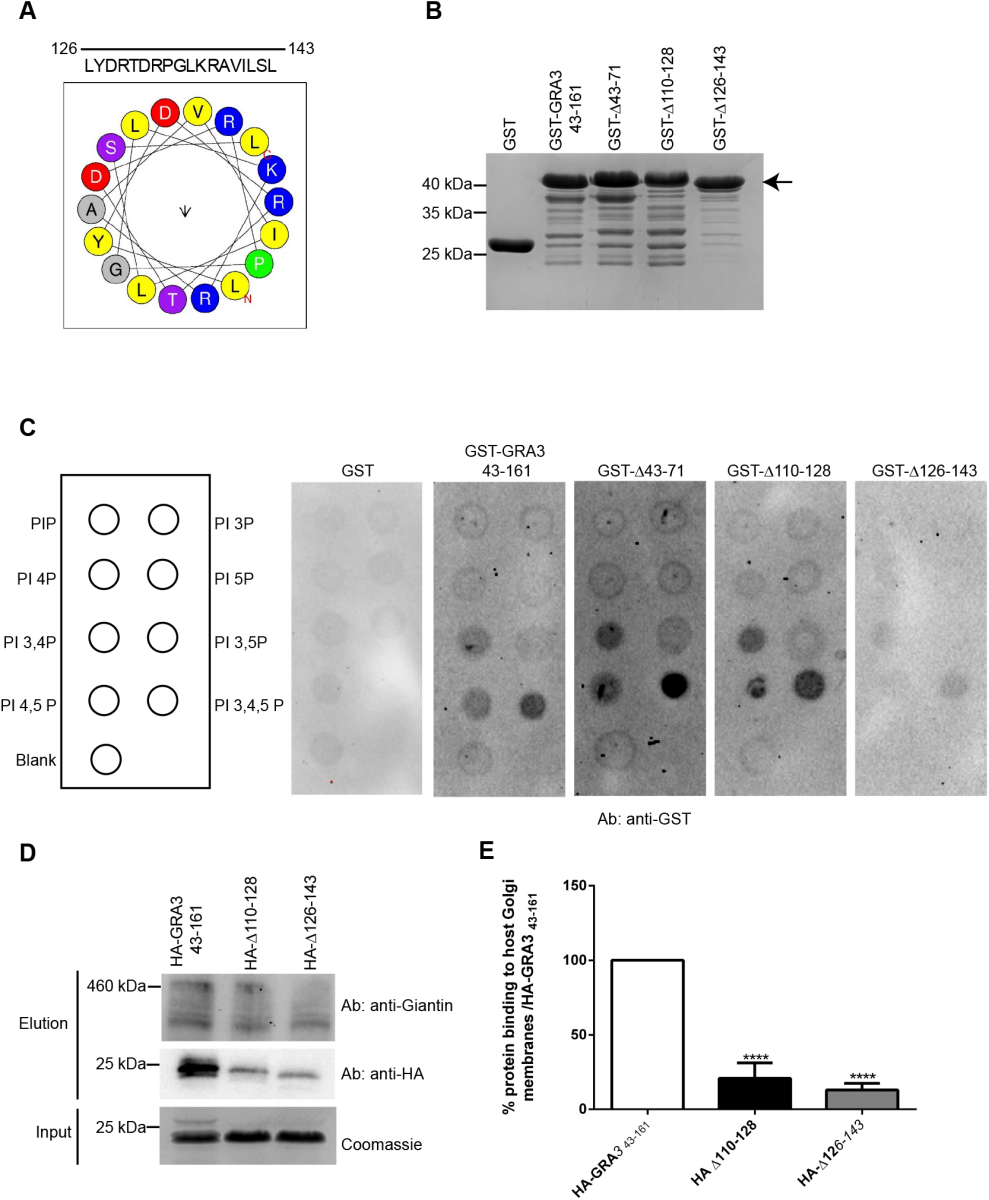
**TgGRA3 is able to bind phosphatidylinositol lipids.** (A) Helical wheel representing the putative lipid-binding site of GRA3. Yellow, hydrophobic regions; blue, positively charged amino acids; red, negatively charged amino acids; purple, polar amino acids. (B) Coomassie Blue staining showing an equal amount of the purified recombinant proteins GST, GST-GRA3_43-161_, GST-Δ110-128, GST-Δ43-71, and GST-Δ126-143 (black arrow indicates recombinant TgGRA3 proteins). (C) Protein-lipid overlay analyzing phosphatidylinositide lipid binding to purified recombinant proteins, shown in panel B. Protein-lipid overlays were carried out using antibodies specific to GST. The same quantities of antibodies and exposure times (900 s) were used for all overlays. (D) Western blots representing host Golgi binding to purified recombinant proteins HA-GRA3_43-161_, HA-Δ43-71 and HA-Δ110-128. Proteins were visualized using specific anti-HA antibodies and anti-giantin for the host Golgi marker. (E) Histogram of data from four independent host Golgi-binding assays, described in panel D. Results were compared with those from HA-GRA3_43-161_ protein (100%) and are represented as means±s.d. ****P<0.0001 (unpaired *t*-test).

Previous work showed that full-length TgGRA3 secreted from extracellular parasites can bind to liposomes *in vitro* (Coppens et al., 2006), but it remained unclear whether lipids and TgGRA3 interact directly. According to calculations done with HeliQuest (Gautier et al., 2008) (Keller, 2015), we identified amino acids 126–143 as a putative TgGRA3 lipid-binding site (Fig. 6A). Hence, we generated a mutant protein lacking this region (GST-Δ126-143) and purified it similarly to recombinant GST alone, GST-GRA3_43-161_, GST-Δ43-71, and GST-Δ110-128 (Fig. 6B). Protein-lipid overlays were performed using similar amounts of purified recombinant proteins in the presence of phospholipids, including lipids mostly present in the host Golgi apparatus [PI-(4)P; Fig. 6C]. We found that GST-GRA3_43-161_, GST-Δ43-71, and GST-Δ110-128 bound specifically to PI-(3,4) P_2_, PI-(4,5) P_2_, and PI-(3,4,5) P_3_, whereas GST-Δ126-143 did not bind these lipids (Fig. 6C). We conclude that the lipid-binding site of TgGRA3 is located between amino acids 126 and 143. Moreover, we found that TgGRA3 binds to lipids enriched at the host plasma membrane PI-(4,5) P_2_ or endosomes PI-(3,4,5) P_3_. As PI-(3,4,5) P_3_ was also found at the host Golgi (Low et al., 2010), we did not exclude its interaction with TgGRA3. Therefore, we decided to directly test whether recombinant TgGRA3 and its corresponding mutant recombinant proteins could bind to host Golgi-enriched membranes *in vitro*. Purified HA-GRA3_43-161_ bound host Golgi-enriched membranes (Fig. 6D), but this interaction was impaired in HA-Δ110-128 (20.81%±10.35%) and HA-Δ126-143 (19.97%±4.47%) (Fig. 6E). These results suggest that the C-terminal region of TgGRA3 may be required for direct interaction with host Golgi.

### TgGRA3 forms a complex with TgGRA23 through its coiled-coil domain

**Fig. 7.**
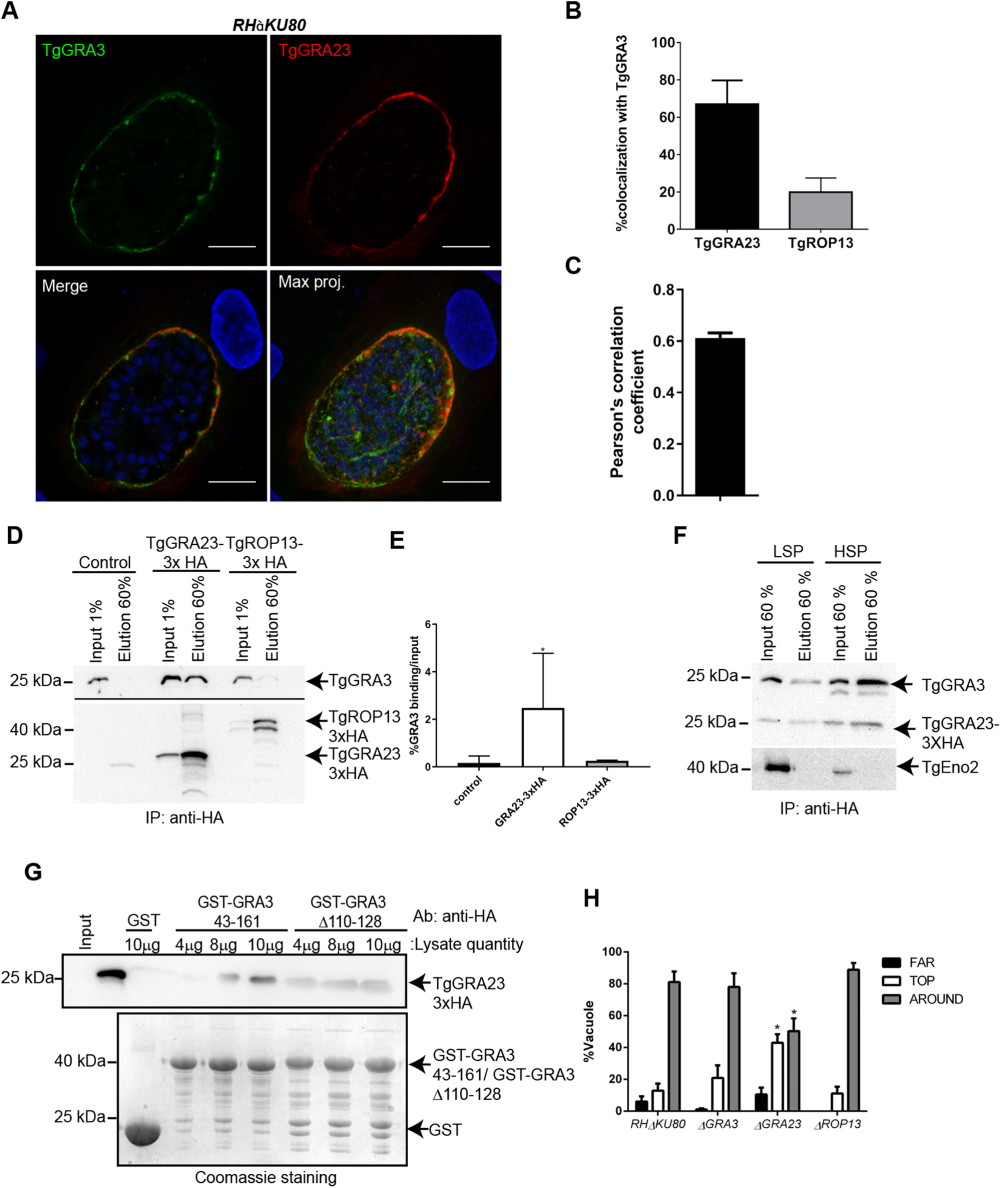
**TgGRA3 interacts with TgGRA23 but not TgROP13.** (A) Representative z-stack images from confocal microscopy showing nuclei (DAPI, blue), TgGRA3 (green), and TgGRA23_3×HA (red). Scale bars: 10 µm. (B) Percentage of colocalization between TgGRA3 and TgGRA23_3×HA or TgROP13_3×HA was measured after three-dimensional reconstruction of z-stack images (*n*=16 parasitophorous vacuoles). Results are reported as means±s.d. (C) Pearson's correlation coefficient from data in B confirmed partial co-localization between TgGRA3 and TgGRA23. Results are reported in±s.d. (D) Western blot representative of three independent experiments showing immunoprecipitation of TgGRA3 by TgGRA23_3×HA. TgGRA23_3×HA and TgROP13_3×HA lysates were incubated with anti-HA antibodies coupled to Sepharose beads, followed by immunoblotting for TgGRA3, TgGRA23_3×HA, and TgROP13_3×HA. (E) Quantification of immunoprecipitation carried out in D. Results from three independent experiments are represented. Results are reported as means±s.d. *P=0.0311 (Welch's *t*-test). (F) Immunoprecipitation of TgGRA23_3×HA in parasite extracts (low-speed supernatant: LSP) and PVM extracts (high-speed supernatant: HSP). TgGRA3 and TgGRA23_3×HA were detected by immunoblotting. The parasite nuclear marker TgENO2 was used as a negative control. (G) Binding of TgGRA23_3×HA to GST-GRA3_43-161_ and GST-Δ110-128 versus GST alone. Bound TgGRA23_3×HA was detected by immunoblotting with anti-HA antibodies. Equal quantities of TgGRA3 proteins were confirmed by Coomassie Blue staining. This blot shows no binding of TgGRA23_3×HA to GST-Δ110-128. (H) Histogram quantifying different host Golgi localizations in cells infected by Δ*GRA23* or Δ*ROP13* parasites versus *RH*Δ*KU80* and Δ*GRA3* parasites. Golgi localizations were classified as not recruited (far), localized above the PV (top) or surrounding the PV (around). For each condition, 300 parasitophorous vacuoles from three independent experiments were counted. *P<0.05 (two-way ANOVA). Results are reported as percentage±s.e.m.

TgGRA3 might not act alone in the host Golgi entry process. Previous studies of TgGRA3 did not identify binding partners for this protein (Dunn et al., 2008; Ossorio et al., 1994). However, the RELYD sequence situated in the TgGRA3 coiled-coil region was identified as a putative HT/PEXEL motif (host-targeting *Plasmodium* export element) (Hsiao et al., 2013). In *Plasmodium falciparum*, the *Pf*EXP2 protein mediates the export of proteins containing the PEXEL (Gold et al., 2015). Recent work demonstrated that TgGRA23 localized at the PVM and shares 22% homology with *Pf*EXP2 (Gold et al., 2015), a putative protein-conducting pore. We hypothesized that TgGRA23 might interact with TgGRA3 through its coiled coil domain that contains the TEXEL (*Toxoplasma* export element) motif. This interaction would require the co-localization of TgGRA3 and TgGRA23 at the PVM. To verify our hypothesis, we created a parasite line expressing a HA-tagged version of TgGRA23, and then used confocal microscopy to observe TgGRA23 localization relative to TgGRA3. Quantification of fluorescence intensities (67.01%±12.76%) supported by Pearson's correlation coefficient measurements (0.61±0.018) revealed a partial colocalization between TgGRA3 and TgGRA23 at the PVM (Fig. 7A–C).

Using immunoprecipitation assays, we noticed that TgGRA3 co-eluted with TgGRA23_3×HA (Fig. 7D–7E). As negative control, we found that another PVM-localized TgROP13_3×HA protein did not co-immunoprecipitate with TgGRA3 (Fig. 7D–E), suggesting that TgGRA23 may specifically bind to TgGRA3. Then, to determine the location for this interaction, we fractionated the PVM from TgGRA23_3×HA-expressing intracellular parasites by differential centrifugation (Fig. 6F). The low-speed pellet (LSP), which contained parasites, and the high-speed pellet (HSP), which contained PVM, served as inputs for immunoprecipitation assays. TgGRA3 and TgGRA23_3×HA co-immunoprecipitated inside the parasite and at the PVM (Fig. 7F), suggesting that TgGRA3 and TgGRA23 formed a complex *in vivo*. The glycolytic enzyme TgENO2, used for quality control of cell fractionation (Ferguson et al., 2002; Mouveaux et al., 2014), exhibited a marginal level of TgENO2 contamination in the input HSP sample (Fig. 7F).

Because we were not able to obtain parasites expressing both TgGRA23_3xHA and the GRA3-Δ110-128 tagged protein, we carried out *in vitro* binding assays using GST-Δ110-128 and GST-GRA3_43-161_ bound to glutathione beads with a protein lysate from parasite expressing GRA23_3xHA. We observed that TgGRA23_3×HA specifically bound to GST-GRA3_43-161_ in a concentration-dependent manner whereas no binding was detected for GST-Δ110-128 or GST alone (Fig. 7G).

We next investigated whether TgGRA23 depletion has an effect on host Golgi localization by counting the cells that exhibited particular host Golgi localizations in relation to the PV at 35 h post-infection. In Δ*GRA23*-infected cells, 45.98%±1.67% of the host Golgi was localized at the ‘top’ of the PV, and 46.7%±7.39% ‘around’ the PV (Fig. 7H), whereas nearly 80% of cells had host Golgi ‘around’ the PV in cells infected with control or ΔGRA3 parasites (Fig. 7H). Thus, we observed a defect in host Golgi localization in Δ*GRA23-*infected cells relative to cells infected with the parental strain.

To determine if TgGRA23 localized inside the PVM invaginations when TgGRA3 expression was down regulated, we created a parasite line expressing a HA-tagged version of TgGRA23 inside the iKD-GRA3 parasites. In the absence of ATc, we noticed that both TgGRA23 and TgGRA3 were accumulated with host Golgi material at the specific regions of the PVM (Fig. S6A) even though TgGRA3 and TgGRA23 exhibited a different pattern of localization inside these invaginations (Fig. S6A, magnified regions). Under ATc pressure, TgGRA23 did not localize anymore with host Golgi accumulated material at the PVM. We then conclude that the presence of TgGRA3 is required for the location of TgGRA23 at these PVM invaginations (Fig. S6B).

We also decided to knockout the Tg*GRA23* gene (Fig. S3C,D). This deletion slightly affected TgGRA3 localization at the PVM, but not in PV projections (Fig. S6C). We were unable to obtain Δ*GRA23-*Δ*GRA3* double mutants to further investigate the possible concomitant role of the two proteins in host Golgi binding. This failure also suggests that the presence of both TgGRA3 and TgGRA23 is essential for parasite viability.

### TgGRA3 disturbs the anterograde pathway of infected host cells

**Fig. 8.**
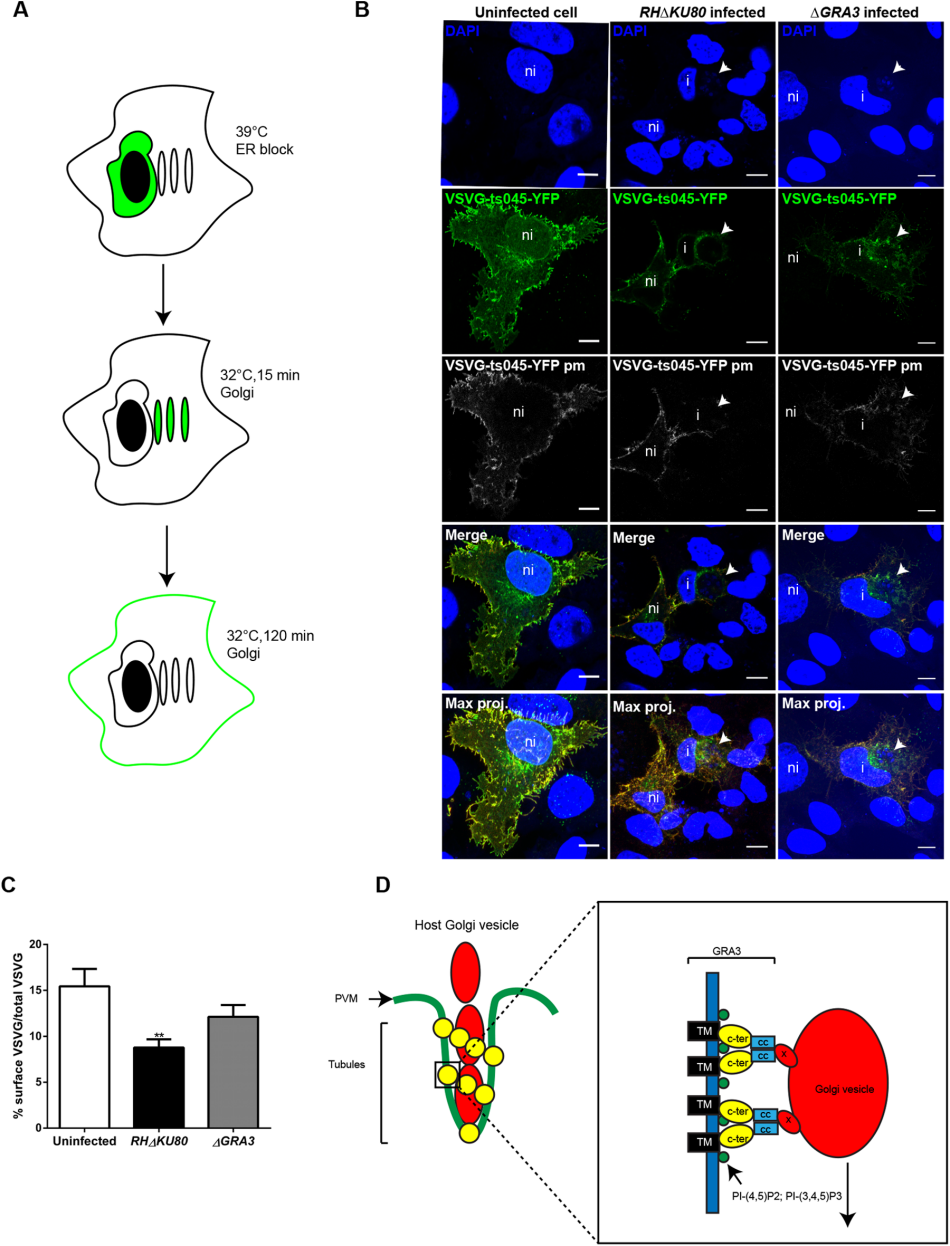
**TgGRA3 regulates the host anterograde pathway.** (A) Schematic of VSVG-ts045-YFP trafficking. Cells were incubated at 39°C to block VSVG-ts045-YFP (green) in the endoplasmic reticulum (ER). Cycloheximide was added to inhibit new protein synthesis, and cells were incubated at the permissive temperature (32°C) to allow VSVG-ts045-YFP trafficking to the Golgi (15 min) and then to the plasma membrane (PM, 120 min). (B) Representative z-stack confocal images showing VSVG-ts045-YFP (green), VSVG-ts045-YFPpm at the plasma membrane (gray), and VSVG-ts045-YFPpm (red for merge and max. proj.). ni, non-infected cell; I, infected cell; white arrow, parasitophorous vacuole; Max. Proj., maximum projection. Brightness of DAPI staining was increased to visualize parasites nuclei (C). Following three-dimensional reconstruction, histogram quantifying the percentage of VSVG-ts045-YFP at the cell surface was normalized by the total VSVG-ts045-YFP after 120 min of incubation at 32°C. Results are represented as percentage±s.e.m. 20 cells from three independent experiments were analyzed. **P<0.001 (unpaired *t*-test). (D) Model depicting the role of TgGRA3 in host Golgi material entry inside the PV. TgGRA3 (yellow) forms oligomers at the PVM (green), leading to formation of tubules containing host Golgi vesicles (red). Right panel (magnified region): TgGRA3 is recruited to specific PI-(4,5) P_2_, PI-(3,4,5) P_3_ rich regions (green) through its lipid-binding domain (yellow). This accumulation facilitates TgGRA3 oligomerization and its interaction with an unknown protein (X) on host Golgi vesicles (red). Concentration of host Golgi material and TgGRA3 could facilitate membrane deformation. The tubules could drive host Golgi vesicle entry inside the PV. CC, coiled-coil domain (blue); C-ter, lipid-binding domain (yellow); TM, transmembrane domain (black).

We hypothesized that if TgGRA3 interacts with host Golgi, this would have an impact on the host anterograde trafficking, which is necessary for protein transport from the Golgi apparatus to the endosomes and plasma membrane. To test this hypothesis, we monitored the trafficking of a temperature-sensitive mutant of vesicular stomatitis virus glycoprotein (VSVG-ts045-YFP) (Fig. 8A,B). At time 0, VG-ts045-YFP is blocked at 39°C in the ER (Fig. S7). At permissive temperature (32°C, 15 min), VG-ts045-YFP was transported to the host Golgi apparatus. While non-infected cells and cells infected by Δ*GRA3* mutants were showing host Golgi localization of VSVG-ts045-YFP, cells infected with *RH*Δ*ku80* exhibited a localization of VSVG-ts045-YFP at the perinuclear region of the ER and at the host Golgi (Fig. S7, yellow arrow).

We measured the amount of VSVG-ts045-YFP that reached the plasma membrane using an antibody that recognizes its extracellular domain. The percentage of VSVG-ts045-YFP reaching the plasma membrane after 120 min was lower in cells infected with *RH*Δ*KU80* parasites (8.77%±0.91%) than in non-infected cells (15.45%±1.90%; Fig. 8C). When Δ*GRA3* mutants were grown in host cells, normal amounts of VSVG-ts045-YFP were delivered at the plasma membrane (12.13%±1.28%) (Fig. 8C). The latter result indicates that intracellular trafficking from the ER via the Golgi to the plasma membrane is impacted in cells infected by *T. gondii*. Therefore, TgGRA3 might have a function in modulating the anterograde pathway through its interaction with host organelles.

## DISCUSSION

In this study, we revealed one of the molecular mechanisms of host organelle recruitment by the parasite *T. gondii*. Combining biochemical approaches and mass spectrometry, we showed that TgGRA3 interacts with the host Golgi apparatus. Previously, yeast two-hybrid studies indicated that TgGRA3 binds to the host ER protein calcium-modulating ligand (CAMLG) (Kim et al., 2008). In addition, a retrieval ER motif was found in TgGRA3 C-terminal sequence, suggesting a role of this protein in host organelle hijacking (Henriquez et al., 2005). However, it was reported that TgGRA*3-*knockout mutants were not impaired in recruitment of host ER and mitochondria (Craver and Knoll, 2007). Instead, we clearly showed that TgGRA3 could bind to host Golgi-enriched fractions. The host Golgi seemed to enter into the PV through TgGRA3 coated invaginations formed at the PVM. We do not know whether TgGRA3 has a direct interaction to host Golgi proteins or lipids or if this binding requires other partners. Nevertheless, we do know that the C-terminal region of TgGRA3 contains a lipid-binding site and a coiled-coil domain that is important for the protein oligomerization. The C-terminal region of TgGRA3 may be required for host Golgi association and binding to another dense granule protein TgGRA23. Based on these findings, we propose that TgGRA3 coats tubules, which are necessary for targeting host organelles to the PV and subsequent membrane scavenging (Fig. 8D).

In addition, we showed that TgGRA3 modulates anterograde trafficking of infected host cells, a function related to the targeting of host Golgi material to the PV. We found that the parasite also secretes a filamentous network containing TgGRA3 into the host cytoplasm. This network connecting the PV is in very close proximity to the host Golgi. TgGRA3-positive filamentous projections were previously observed in *T. gondii* (Dubremetz et al., 1993) and *Plasmodium chabaudi* (Lanners et al., 1999), and similar PV projections have been observed in association with TgGRA7 (Dunn et al., 2008; Romano et al., 2013). The composition and role of these projections remain unknown but they could be involved in tethering host organelles including the Golgi apparatus to the PV. Interestingly, filaments originating from vacuoles containing intracellular bacteria such as *Salmonella* have also been observed and are thought to be important for bacterial replication (Garcia-del Portillo et al., 1993). We hypothesize that, in *T. gondii*, these filaments are important for delivery of proteins involved in host Golgi fragmentation or targeting of organelles from other host vesicles.

Parasites lacking TgGRA3 also had a defect in host Golgi material entry at the PVM. This result reveals that TgGRA3 may play dual roles: one in host Golgi targeting to the PV and one in Golgi material entry. Indeed, we observed that TgGRA3 coats tubules wrapping host Golgi vesicles in the intravacuolar network. Moreover, TgGRA3 could also influence the shape of these tubules in the intravacuolar network, as evidenced by the abnormal vesicles in TgGRA3-depleted parasites, a phenotype not reported previously (Rommereim et al., 2016). In addition, their disappearance in TgGRA3 knockdown parasites indicated a role of TgGRA3 in their maintenance. Observation of INV tubules fusing with the PVM are not uncommon and were previously visualized with host microtubules (Coppens et al., 2006). Although, we still could not determine if this INV tubules are acting as a common mechanism used by *T. gondii* to consume host organelles. Purified recombinant TgGRA3 binds lipids and oligomerizes due to its C-terminal region. TgGRA3 binds specifically to PI-(3,4,5) P_3_, which was found at the host Golgi (Low et al., 2010) but also PI-(3,4) P_2_ and PI-(4,5) P_2_ lipids, which are principally found in the host plasma membrane. Given that the PV originates from the host plasma membrane, and considering the proximity of the lipid-binding site with one of TgGRA3 putative transmembrane domain, we suggest that these lipid species may form microdomains at the PVM, which can recruit TgGRA3 to the PVM. Accordingly, we propose a model for TgGRA3 function in host cell Golgi recruitment that requires protein localization to specific lipid domains of the PVM and oligomerization (Fig. 8D). Crystal structure studies on TgGRA3 protein would be necessary to verify TgGRA3 topology and support our finding.

It has been described that intracellular *T. gondii* sequesters host Golgi Rab vesicles via a phagocytosis-like process and tubules containing two other dense granule proteins, TgGRA2 and TgGRA6 (Romano et al., 2017). This observation confirms the roles played by dense granule proteins in sequestration and engulfment of host organelles. The functional redundancy of these proteins may explain why knockout of only one gene does not have severe deleterious consequences on parasite survival. We conclude that these dense granule proteins, including TgGRA3, act in concert to promote sequestration of host organelles, thereby providing nutrients essential for parasite growth. In addition, we propose that TgGRA3, which binds to specific lipid domains, could cause PVM deformation and modulate the formation of tubules. Host Golgi material interacts with TgGRA3 at these sites and then could induce its own transport inside TgGRA3-coated tubules.

Finally, we observed that depletion of TgGRA3 restored a normal anterograde trafficking in infected host cells, suggesting that the parasite could regulate the host secretory pathway. This observation indicates that, in addition to lipid scavenging, the parasite could recruit host Golgi membranes for other reasons. The ability of *T. gondii* to divert the host cell's Rab positive vesicles that are involved in anterograde trafficking (Romano et al., 2013) is in good agreement with our finding. The potential advantages of recruiting host Golgi membranes and modulating anterograde transport could include inhibition of cytokine secretion and the host immune response, as previously proposed by Murray and Stow (2014). As mentioned above, TgGRA3 also plays an important role in parasite virulence (Craver and Knoll, 2007), a phenotype that may be linked to the pre-emption of key host immune molecules involved in controlling the outcome of parasite infection. In line with this point of view, proteomic studies identified TgGRA3 as a protein that was more highly expressed in virulent *T. gondii* strains than in less virulent strains (Qiu et al., 2016).

In conclusion, this study reveals a new strategy employed by *T. gondii* to target the host Golgi apparatus and divert its functions. Different parasite mutants, including those lacking multiple dense granule proteins (e.g. simultaneous knockouts of TgGRA2, TgGRA3, and TgGRA6 or others), may completely abrogate host Golgi binding and recruitment. These kind of mutants would provide deeper insight into the role of host organelle hijacking by *T. gondii*.

## MATERIALS AND METHODS

### Host cell culture

Human foreskin fibroblast (HFF) cells were maintained in Dulbecco's modified Eagle's medium (DMEM, GIBCO, Invitrogen, France) supplemented with medium A (10% fetal calf serum, 0.5 mg/ml penicillin-streptomycin, and 2 mM L-glutamine). Chinese hamster ovary (CHO-K1) cells were maintained in F12-K nutrient mixture with medium A. Cells were cultured at 37°C in 5% CO_2_.

### Plasmids

Table S3 lists all primers used during this work. The ligation independent cloning (LIC) method was used to generate these parasite lines by integrating genes encoding 3xHA-tagged version of Tg*ROP13* and Tg*GRA23* into the respective endogenous open reading frames by single homologous recombination. The knock-in parasites pLIC-GRA23_3xHA-*dhfr*, pLIC-ROP13_3xHA-*dhfr* were created by amplifying their respective genomic coding sequences. The Δ*GRA3* mutant was created using primers KOGRA3_5UTR_F and KOGRA3_5UTR_R for the 5′ flanking region and KOGRA3_3UTR_F and KOGRA3_3UTR_R for the 3′ flanking region. The Δ*GRA23* mutant was created using primers KOGRA23_5UTR_F and KOGRA23_5UTR_R for the 5′ flanking region and KOGRA23_3UTR_F and KOGRA23_3UTR_R for the 3′ flanking region.

The PG13D-T7-S4 GRA3 plasmid driven by the inducible promoter tetO7SAG4 (Sheiner et al., 2011) was created using the primers PDTS4KOGRA3_3UTR_F and PDTS4KOGRA3_3UTR_R for the 3′ flanking region and the primers PDTS4KOGRA3_5UTR_F and PDTS4KOGRA3_5UTR_R for the 5′ flanking region. RH genomic DNA was used as a PCR template.

For expression of the recombinant protein GST-GRA3_43-161_ in *E. coli*, DNA fragments corresponding to amino acids 43–161 were amplified by PCR from parasite cDNA. The amplified DNA was cloned in-frame with GST at the *Bam*HI restriction site of pGEX6-P3 (GE Healthcare) with primers pGEX6-P3_GRA3rec_F and pGEX6-P3_GRA3rec_R. GST-HA-GRA3_43-161_ wild type was generated with primer HA-GRA3_F. Mutants GST-Δ110-128 and GST-HA-Δ110-128 were created by PCR using primers Δ110-128_F and Δ110-128_R. Mutants GST-Δ43-71 and GST-HA-Δ43-71 were created by PCR using primers Δ43-71_F and Δ43-71_R. Mutants GST-Δ126-143 and GST-HA-Δ126-143 were generated by PCR using primers Δ126-143_F, Δ126-143_R, Δ110-143_F, and Δ110-143_R.

### Parasite culture and drug selection

*Toxoplasma gondii RH*Δ*ku80* or *RHTaTi* parental and transgenic strains were maintained in confluent HFF cells using medium A. For parasite transfection, 50 µg of plasmid DNA was linearized and electroporated using 2 mm cuvettes (Eurogentec, France). Parasites were cultured in the presence of 2 µg/ml pyrimethamine to select for the dihydrofolate reductase (*DHFR*) resistance cassette, 25 µg/ml mycophenolic acid and 50 µg/ml xanthine for the hypoxanthine-xanthine guanine phosphoribosyl transferase (*HXGPRT*) resistance cassette, and 5 µM 5-fluorodeoxyuridine (FUDR) for pUPRT selection. Parasites containing the tetracycline-regulated gene expression were pre-treated with 1.5 µg/ml of anhydrotetracycline (ATc) for 48 h then used to infect new HFF cells in presence of ATc 1.5 µg/ml for 30–48 h.

### Cell fractionation

CHO-K1 cells were cultured in 20 T-175 flasks. Cells were washed three times with PBS and incubated in hypo-osmotic buffer (10 mM HEPES, pH 7.4) for 10 min. Cells were then scraped in SEAT buffer (250 mM sucrose, 1 mM EDTA, 10 mM acetic acid, 10 mM triethanolamine, pH 7.4) and centrifuged at 500 ***g*** for 5 min. The supernatant was collected and subjected to centrifugation at 100,000 ***g*** for 30 min. The membrane pellet was suspended in 15% sucrose. Sucrose gradients were generated as previously described (Radhakrishnan et al., 2008).

### Preparation of parasite lysate

Freshly egressed parasites were lysed by five freeze-thaw cycles in liquid nitrogen. This method resulted in release of some organelle contents, such as rhoptries, dense granules and micronemes, into the cytosolic fraction. The lysate was spun at 500 ***g*** for 5 min at 4°C to remove the parasites and parasite debris. The resultant supernatant was subjected to centrifugation at 100,000 ***g*** for 30 min at 4°C to collect soluble fractions.

### Golgi-binding assay

Golgi membranes (200 µg) were incubated with 1 mg/ml parasite lysate in buffer A (10 mM HEPES pH 7.4, 150 mM NaCl, 1 mM GTP, 1 mM ATP, 250 mM sucrose, and protease inhibitor cocktail (Thermo Fisher Scientific, France) for 30 min at 37°C. The membranes were pelleted at 100,000 ***g*** for 30 min and washed three times with buffer A to remove unbound proteins. Membranes containing parasite proteins were lysed in Laemmli buffer (50 mM Tris pH 7.4, 150 mM NaCl, 0.2% SDS, 100 mM DTT, and 10% sucrose) and processed for mass spectrometry or western blotting. For experiments with GRA3_43-161_, Δ126-143, and Δ110-143, 40 µg of Golgi membranes was incubated with 10 µg of purified proteins for 30 min at 37°C.

### Protein identification by mass spectrometry

Protein digests were analyzed using C18 reverse-phase columns on an Ultimate 3000 RSLCnano System (Dionex/Thermo Fisher Scientific) coupled to a Q-Exactive mass spectrometer (Thermo Fisher Scientific) as previously described (Sangaré et al., 2016). Briefly, eluted peptides from the C18 analytical column were analyzed on a Q-Exactive mass spectrometer; for ionization, a nanospray Flex Ion Source was used at a voltage of 1.9 kV and a capillary temperature of 275°C. Parameters of the acquisition method were as follows: full MS scans were acquired in the Orbitrap mass analyzer over the *m/z* 300–1800 range with a resolution of 70,000 at *m/z* 200. A target automatic gain control (AGC) value of 5×10^5^ was used with a maximum allowed injection time (Maximum IT) of 250 ms. For MS/MS, an isolation window of 2 m*/z* was used. The ten most intense peaks (TopN) with charge state between 2 and 4 were selected for fragmentation in the higher-energy collisional induced dissociation (HCD) cell, with normalized collision energy of 27. The tandem mass spectra were acquired over the *m/z* 200–2000 range in the Orbitrap mass analyzer with resolution 35,000 at *m/z* 200 and an AGC of 5×10^4^. The ion intensity selection threshold was 1.7×10^4^, and the maximum injection time was 150 ms. Dynamic exclusion was set to 20 s (15 s for a long gradient). The total run time was 60 min for a short gradient and 120 min for a long gradient. All systems were fully controlled by Thermo Xcalibur 3.0 (Thermo Fisher Scientific).

For protein identification, all data files (*.raw) collected during nanoLC-MS/MS analyses were processed with a specific workflow designed in Proteome Discoverer 1.4 (Thermo Fisher Scientific) as described (Sangaré et al., 2016). Searches with a mass tolerance of 10 ppm for precursor ions and 0.02 Da for fragment ions were performed against two different databases corresponding to the sample: *T. gondii* protein sequences were downloaded from www.toxodb.org on December 11, 2014 (18,954 entries), and *Cricetulus griseus* protein sequences (34,841 entries) were downloaded from Universal Protein Resource (UniProt) on September 29, 2014. The target-decoy database search allowed the estimation of the false-positive identification rate of the present study (Elias and Gygi, 2007). The final catalogue of proteins has an estimated false-positive rate <1%.

### Immunoprecipitation

Parasites (50×10^6^ tachyzoites) from pLIC GRA23_3×HA-*dhfr* and pLICROP13_3×HA-*dhfr* strains were lysed in immunoprecipitation buffer B (10 mM HEPES, pH 7.4, 1.5 mM MgCl_2_, 10 mM KCl, 0.5 mM DTT, 0.1 mM EDTA, 0.65% NP-40, and 0.5 mM PMSF), and then incubated on ice for 20 min. The lysate was pre-cleared by centrifugation at 10,000 ***g*** for 30 min, and the resultant supernatant was incubated overnight at 4°C with anti-HA beads (Thermo Fisher Scientific). The beads were washed three times with buffer B, and then eluted in Laemmli buffer.

### Intracellular parasites and PVM extraction

PVM extraction was carried out as previously described (Ossorio et al., 1994). Briefly, HFF cells infected with parasites for 24 h were scraped and harvested. The lysate was subjected to low-speed centrifugation to pellet the parasites (700 ***g*** for 5 min). The low-speed supernatant was then subjected to high-speed centrifugation to pellet the PVM (100,000 ***g*** for 30 min). Both pellets were suspended in immunoprecipitation buffer B.

### Western blotting

TgGRA3 was detected with rabbit anti-GRA3 antibody (1:500 dilution), which was a gift from Jean-Francois Dubremetz (University of Montpellier, France). HA-tagged proteins were detected with rat anti-HA (1:1000 dilution, Roche, France) or rabbit anti-HA (1:1000 dilution, Cell Signaling-Ozyme, France). Sucrose gradients were analyzed with rabbit anti-giantin (1:1000 dilution, Abcam, France) and rabbit anti-calnexin (1:1000 dilution, Abcam, France), and rabbit anti-GST (1:1000) and anti-ENO2 (1:1000) (Mouveaux et al., 2014). Secondary antibodies were goat anti-rabbit IgG (1:5000 dilution, Thermo Fisher Scientific) or horseradish peroxidase-conjugated rabbit anti-rat IgG (1:2000 dilution, Thermo Fisher Scientific).

### Fluorescence staining of cells and confocal microscopy

HFF cells were infected with parasites for 35–40 h. After infection, the cells were fixed with 4% paraformaldehyde and 0.02% glutaraldehyde for 20 min. The cells were then permeabilized with 0.1% Triton X-100, 5% FBS in PBS (pH 7.4) at room temperature for PVM observation. For INV observation, cells were permeabilized with Triton X-100 at 0.1% at 37°C. Primary antibodies were used at dilutions of 1:500 for rabbit or mouse anti-GRA3, 1:1000 for rabbit anti-giantin (Abcam), 1:2000 for rabbit anti-GCC185 (a gift from Dr Suzanne Pfeffer), 1:500 for rat anti-HA (Roche), 1:1000 for rabbit anti-HA (Cell Signaling Technology-Ozyme), and 1:500 for mouse anti-GRA5 (BIOTEM, France). All secondary antibodies (Invitrogen, France) were used at dilutions of 1:1000: goat anti-mouse-Alexa Fluor 488, goat anti-rabbit-Alexa Fluor 647, goat anti-rabbit-Alexa Fluor 594, and goat anti-rat-Alexa Fluor 488. Cells were imaged in z-stacks (z=0.389 µm) on Zeiss LSM 780, 880 confocal microscopes at 63× magnification. For quantification of host Golgi orientation, mosaics of 49 fields were generated using a Zeiss Apotome microscope.

### YFP-VSVG transport assay

HeLa cells were infected with *RH*Δ*KU80* or Δ*GRA3* parasite strains 6 h before transfection. The cells were transfected (FuGENE HD) with VSVG-ts045-YFP (kindly provided by Dr Suzanne Pfeffer) and incubated at 39°C overnight (5% CO_2_) to block ER exit. After 16 h, 100 μg/ml cycloheximide (Sigma-Aldrich, France) was added to prevent new synthesis of VSVG-ts045-YFP. The cells were then incubated at 32°C for 15 or 120 min to release ER-accumulated VSVG-ts045. The cells were fixed with 4% paraformaldehyde for 20 min and incubated for 1 h in culture supernatant containing VSVG antibodies (kindly provided by Dr Suzanne Pfeffer). Goat anti-mouse-Alexa Fluor 647 (Invitrogen) was used as the secondary antibody. Cells were imaged in z-stacks at 63× magnification on a Zeiss LSM 880 scanning confocal microscope as described above.

### Electron microscopy

Ultrastructural imaging was performed as previously described (Olguin-Lamas et al., 2011). Briefly, HFF cells were infected for 30 h with parental *RH*Δ*KU80* parasites or Δ*GRA3* mutants. Cells were fixed in 2.5% glutaraldehyde prepared in 0.1 M cacodylate buffer and post-fixed in 1% osmium tetroxide in the same buffer. After acetonitrile dehydration, the pellets were embedded in Epon. Serial thin sections (90 nm) were cut using a Leica UC7 ultramicrotome and collected on 150 mesh copper grids. After staining with 2% uranyl acetate in 50% ethanol and incubation with lead citrate solution, sections were observed on a Hitachi H-600 transmission electron microscope at 75 kV, as described (Olguin-Lamas et al., 2011).

### Recombinant protein purification

BL21 bacterial strains were transformed with plasmids encoding GST-GRA3_43-161_ or mutant proteins, and expression was induced with 0.5 mM IPTG for 20 h at 25°C. Bacteria were harvested and suspended in buffer C (50 mM Tris, 150 mM NaCl, 1 mM EGTA, 1 mM EDTA, 1 mM DTT, and 0.5 mM PMSF). Lysozyme (Sigma-Aldrich) was added at 1 mg/ml for 30 min on ice, and then the cells were sonicated. The lysate was incubated overnight with glutathione-Sepharose beads (GE Healthcare, France). Proteins were eluted with 10 mM glutathione in buffer B (50 mM Tris, pH 8, 150 mM NaCl, 1 mM DTT, and 0.5 mM PMSF) or cleaved with PreScission protease (GE Healthcare) in buffer D (50 mM Tris, pH 8, 150 mM NaCl, 1 mM DTT, 0.5 mM PMSF, and 1 mM EDTA). Before assays, purified proteins were spun down at 14,000 ***g*** for 30 min at 4°C to remove protein aggregates.

### *In vitro* binding assay between TgGRA3 recombinant protein and TgGRA23-3xHA parasite lysate

Extracellular parasites modified with pLIC-GRA23_3×HA-*dhfr* were used for protein lysate preparation*.* The parasites were filtered through a 3-μm filter (Whatman, France) and harvested by centrifugation (700 ***g***, 15 min). The parasites were lysed in immunoprecipitation buffer B. pLIC-GRA23_3×HA-*dhfr* lysate was pre-cleared by centrifugation at 14,000 ***g*** for 20 min, followed by incubation with 60 μg of recombinant protein bound to glutathione-Sepharose beads. After incubation at room temperature for 1 h, the beads were washed three times with immunoprecipitation buffer B, suspended in Laemmli buffer, and subjected to SDS-PAGE.

### Protein-lipid overlay assay

Phosphatidylinositol lipids (200 pmol/µl) suspended in 2:1:0.8 methanol:chloroform:water (Echelon Bioscience, France) were spotted onto nitrocellulose membranes (Hybond C Extra, GE Healthcare). The membranes were dried for 1 h at room temperature and blocked for 1 h in buffer E (50 mM Tris, pH 8, 150 mM NaCl, 0.1% ovalbumin, and 0.01% Tween 20). Freshly purified protein was diluted in buffer E to a concentration of 500 µM and incubated overnight with the membranes. The membranes were washed, and classical immunoblots were performed as described above. Rabbit anti-GST (1:1000 dilutions) and HRP goat anti-rabbit (1:5000 dilutions) sera were used.

### Native PAGE

Proteins purified from *Escherichia coli* were captured on glutathione-Sepharose beads. The beads were then incubated for 12 h with PreScission protease (GE Healthcare) in buffer F (25 mM Tris, pH 8, 1 mM DTT, 0.5 mM PMSF, and 1 mM EDTA). After 24 h, the proteins were collected and loaded on a 10% Tris-glycine native gel.

### Size-exclusion chromatography

Recombinant GST-GRA3_43-161_ was cleaved from the GST using PreScission protease as described above. The supernatant containing the cleaved protein was collected and spun at 14,000 ***g*** for 20 min. GRA3_43-161_ was then applied to a Superdex 200 Increase 3.2/300 column using an AKTA Micro FPLC (GE Healthcare). Protein standards (Gel Filtration Marker Kit for Protein Molecular Weights 12,000-200,000, Sigma-Aldrich) were used to generate a standard curve to determine the molecular weight of GST-GRA3_43-161_.

### Reducing and denaturation of TgGRA3 recombinant proteins

Recombinant GST-GRA3_43-161_ was cleaved from the GST using PreScission protease as described above. The supernatant containing the cleaved protein was collected and spun down at 14,000 ***g*** for 20 min. For DTT treatment, GRA3_43-161_ was incubated with 10 mM DTT at 60°C for 15 min. The sample was cooled down at room temperature for 15 min. Iodoacetamide (20 mM) was added, and the sample was incubated for 60 min at room temperature. For urea denaturation, GRA3_43-161_ was incubated with 8 M urea at room temperature.

### Bioinformatics and modeling

All histograms were plotted with GraphPad version 6 (GraphPad Prism, San Diego, USA). Surface three-dimensional reconstructions, volume measurement and quantification of co-localization were performed with Imaris version 8, Imaris XT, Bitplane Inc. (USA). Confocal images were processed using Carl Zeiss Zen software. AutoQuant X3 Version 3.0.5. software was used to perform deconvolution of imaging data. Helical wheel plots were generated using software available online (http://kael.net/helical.htm). Venn diagrams were created using software available online (http://bioinformatics.psb.ugent.be/webtools/Venn/). Coiled-coil predictions were generated by COILS (Lupas et al., 1991). Theoretical modeling of GRA3 protein structure was performed by HHpred and SWISS-MODEL. GRA3 putative secondary structure was predicted with the SOPMA software (Combet et al., 2000). HeliQuest (http://heliquest.ipmc.cnrs.fr/) was used for determination of the putative lipid binding site.

## Supplementary Material

Supplementary information
