# Supplementary material for: The Toxoplasma gondii dense granule protein TgGRA3 interacts with host Golgi and dysregulates anterograde transport
Source: Biol Open. 2019 Feb 27;8(3):bio039818. doi: 10.1242/bio.039818 (PMC6451337; doi:10.1242/bio.039818)
Supplement: Supplementary information [file biolopen-8-039818-s1.pdf]

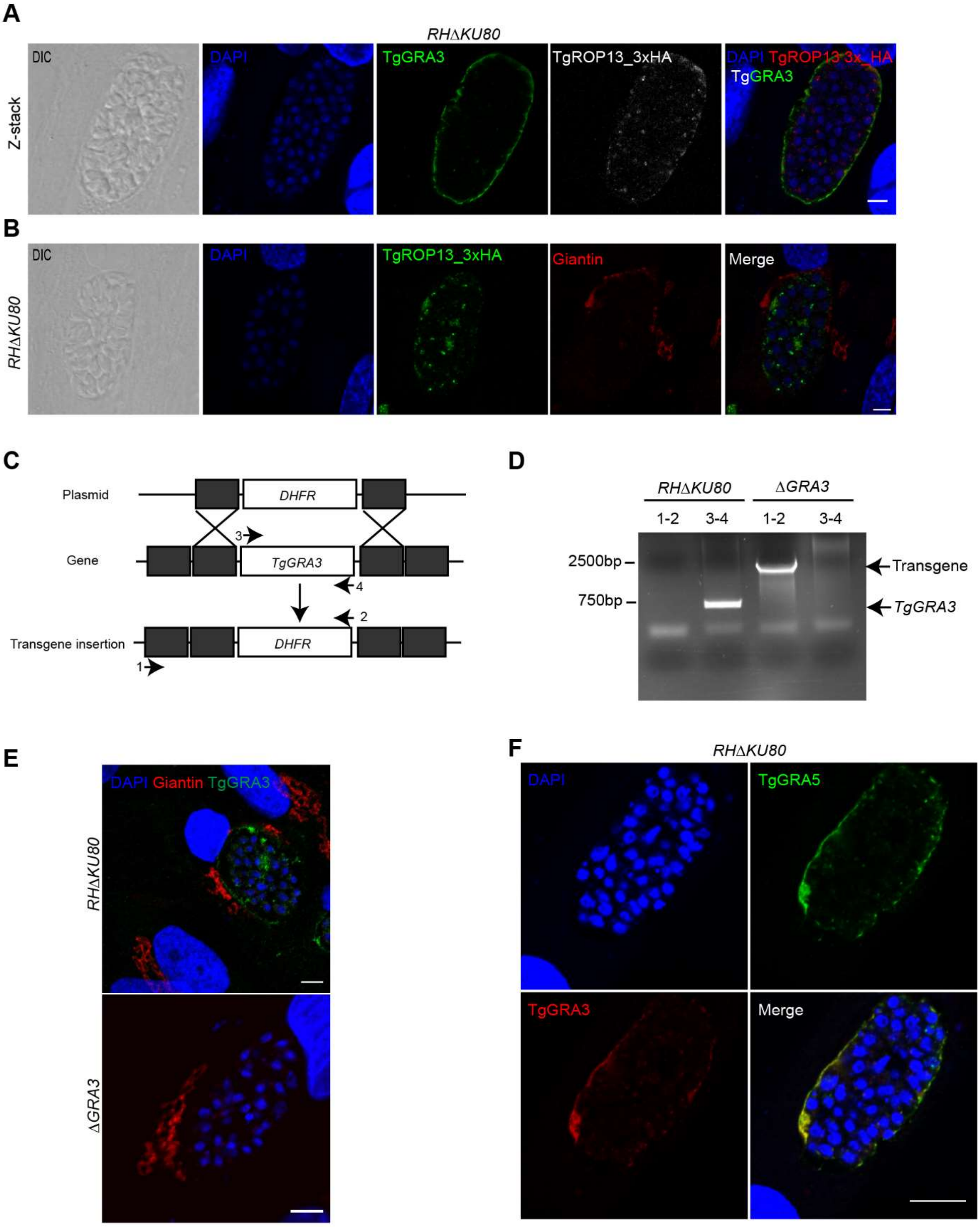
A
RHΔKU80
Z-stack
DIC
DAPI
TgGRA3
TgROP13_3xHA
DAPI TgROP13 3x_HA TgGRA3
B
RHΔKU80
DIC
DAPI
TgROP13_3xHA
Giantin
Merge
C
Plasmid
DHFR
Gene
TgGRA3
Transgene insertion
DHFR
1
2
3
4
D
RHΔKU80
ΔGRA3
1-2
3-4
1-2
3-4
2500bp
750bp
Transgene
TgGRA3
E
DAPI Giantin TgGRA3
RHΔKU80
ΔGRA3
F
RHΔKU80
DAPI
TgGRA5
TgGRA3
Merge

Fig. S1. TgROP13 localization and generation of ∆*GRA3* knock-out mutant

(A) Representative z-stack images from confocal microscopy showing TgROP13_3xHA (gray or red) localization at the PVM. The PVM was labeled with GRA3 (green), and nuclei with DAPI (blue). Scale bars, 10 µm. (B) Representative z-stack images from confocal microscopy indicated the absence of colocalization between TgROP13_3xHA (green) and Giantin (red). Nuclei are stained with DAPI (blue). Scale bars, 10 µm. (C) Approach used for plasmid integration during conditional ablation of Tg*GRA3* gene. (D) Agarose gel showing the disruption of the *TgGRA3* gene and presence of the transgene. (E) Immunofluorescence images confirming depletion of *TgGRA3* protein in ∆*GRA3* knock out mutants. Nuclei are represented by DAPI (blue), TgGRA3 (green) was labeled with anti-TgGRA3 antibody, and Giantin (red) with anti-Giantin antibody. Scale bars, 10 µm. (F) Representative z-stack images from confocal microscopy indicating similar localization between TgGRA3 (red) and TgGRA5 (green). Nuclei are stained with DAPI (blue). Scale bars, 10 µm.

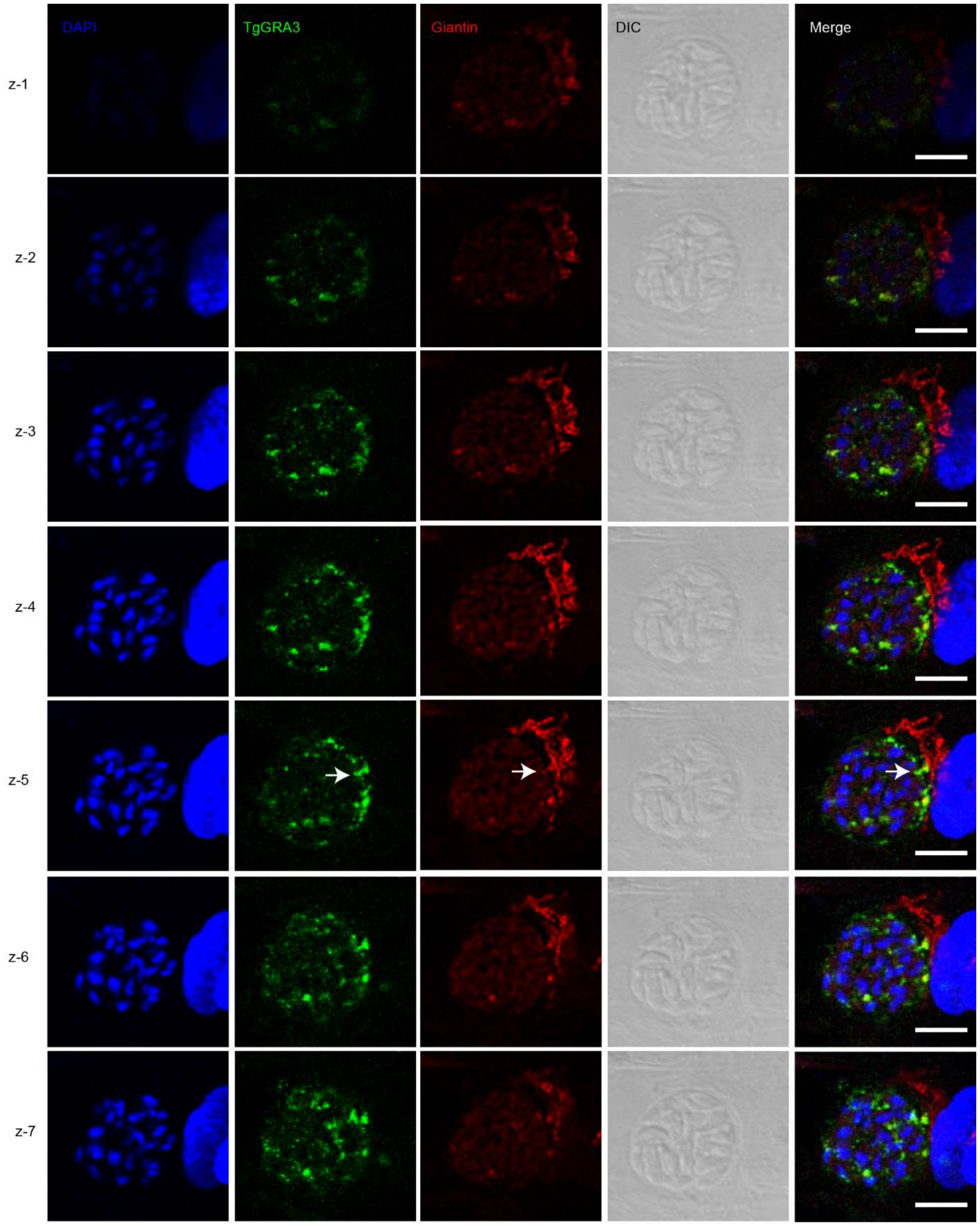

Fig. S2. TgGRA3 tubules are localized inside the PV

Different Z-stack images from Fig.2C were represented from the top view (z-1, first row) to the bottom view (z-7, last row). White arrows indicated TgGRA3 (green) signal colocalizing with Giantin staining (red). Z steps were 500nm apart. Nuclei are stained using DAPI (blue), the vacuole was visualized with DIC. Scale bars, 10 µm.

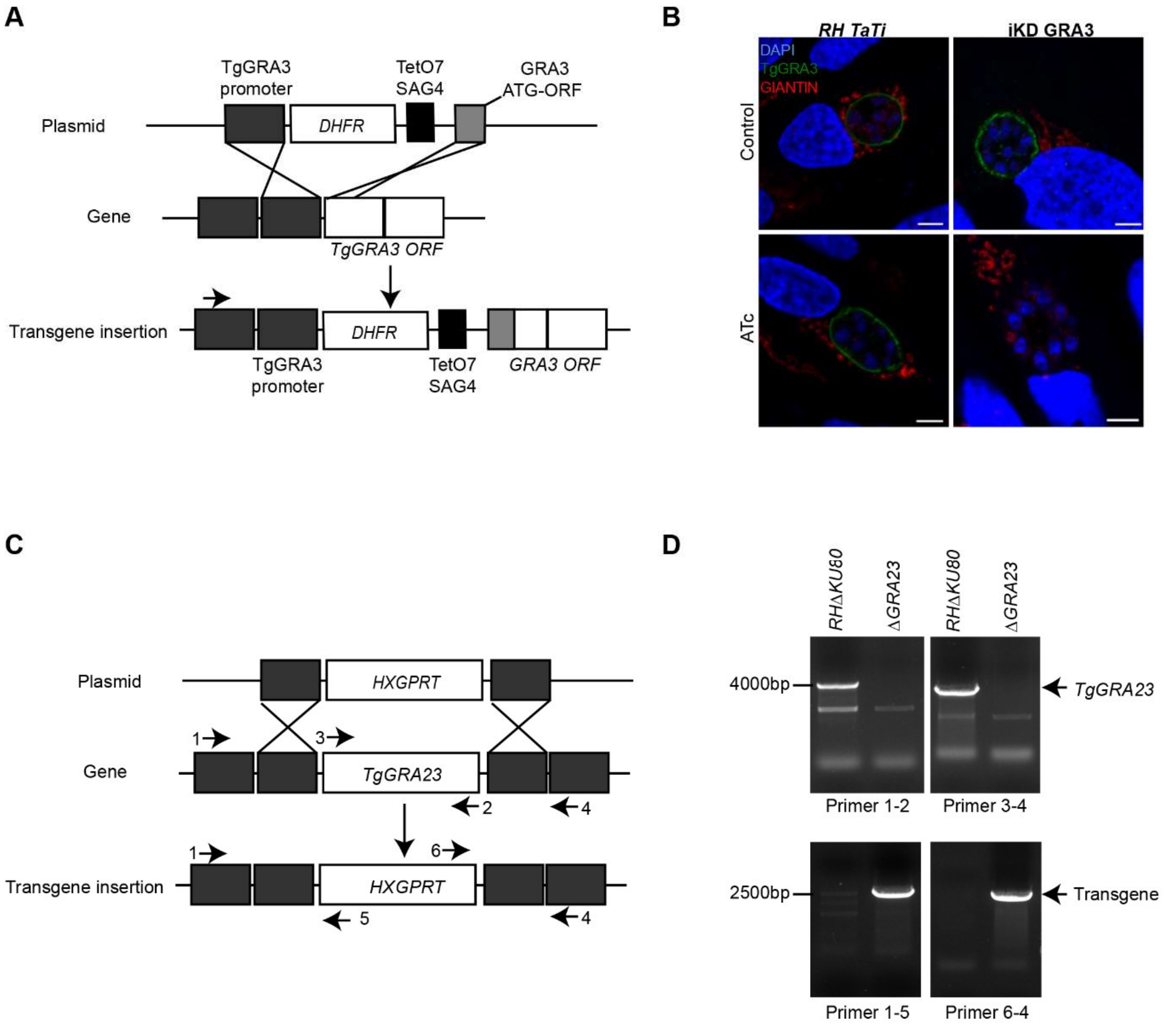

Fig.S3. Generation of the iKD-GRA3 parasites and Δ*GRA23* knock-out parasites

(A) Schematic of the approach used to introduce the transgene inside the *TgGRA3* open reading frame (ORF). (B) Immunofluorescence indicated inhibition of TgGRA3 expression

Biology Open • Supplementary information

48 h after ATc treatment in the iKD-GRA3. TgGRA3 was labeled with anti-GRA3 antibody (green), the host Golgi with the anti-Giantin (red), and nuclei with DAPI (blue). Scale bars, 10 µm. (C) Schematic of the approach used to deplete the Tg*GRA23* gene using the HXGPRT cassette. Arrows represented the position of the numbered primers. (D) Agarose gel showing depletion of the *TgGRA23* gene and introduction of the transgene. Primer numbers referred to labels given in (C).

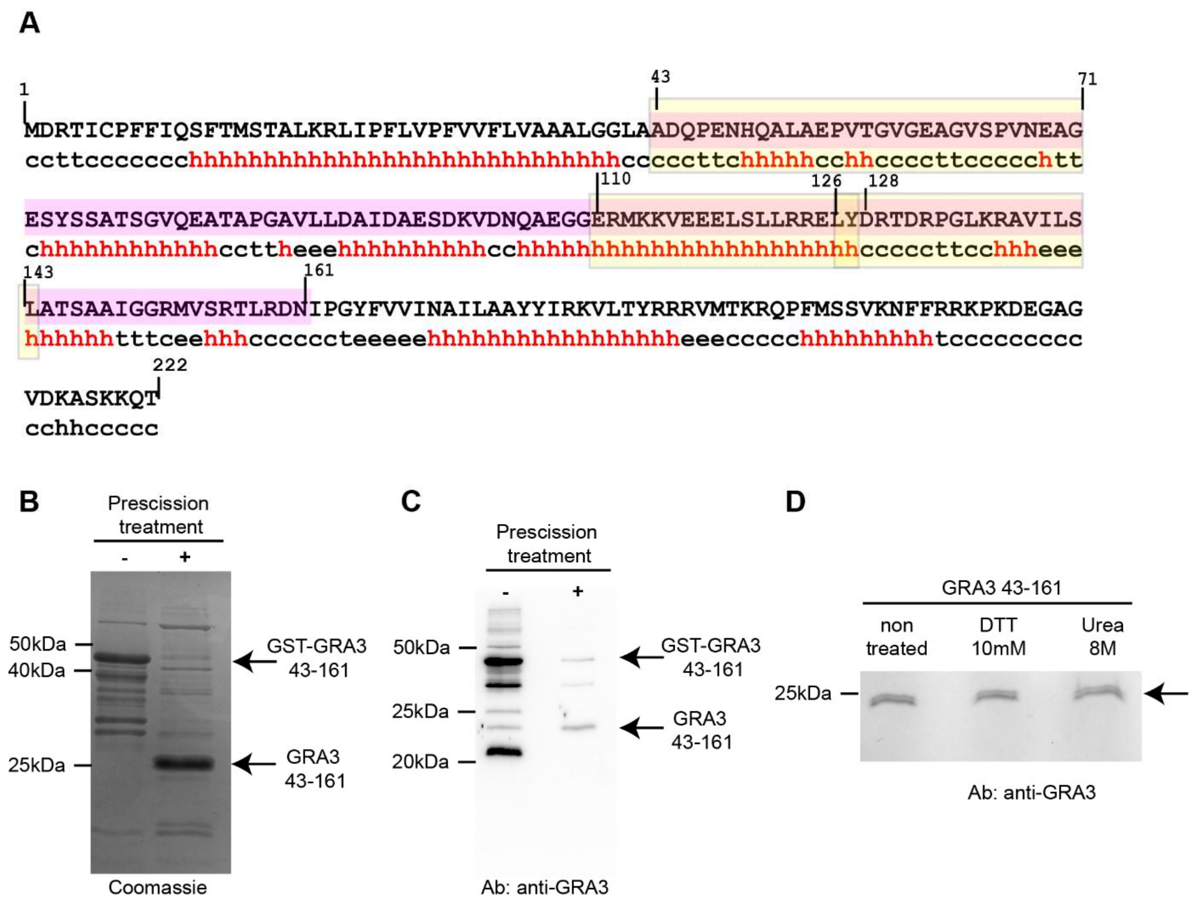

Fig. S4. TgGRA3 biochemical analysis

(A) Secondary structure prediction of TgGRA3 established by SOPMA. The amino acid composition used for the recombinant GRA3$_{43\text{-}161}$ was indicated in pink. Regions (43-71, 110-128, 126-143) deleted from GRA3$_{43\text{-}161}$ recombinant protein were represented in yellow. Secondary structure predictions were mentioned below GRA3 amino acid

Biology Open • Supplementary information

sequence. c:random coiled, h:alpha helix (red), t: beta turn, e: extended strand. (B) Coomassie blue staining of GST-GRA3$_{43\text{-}161}$ purified protein untreated or treated with PreScission. (C) Western blot analysis of GST-GRA3$_{43\text{-}161}$ purified protein untreated or treated with PreScission. GST-GRA3$_{43\text{-}161}$ or cleaved GRA3 $_{43\text{-}161}$ were identified with an antibody specific to TgGRA3. (D) Western Blot of GRA3$_{43\text{-}161}$ treated with 10mM DTT or 8M urea showed no change in the protein mobility on SDS denaturing gel.

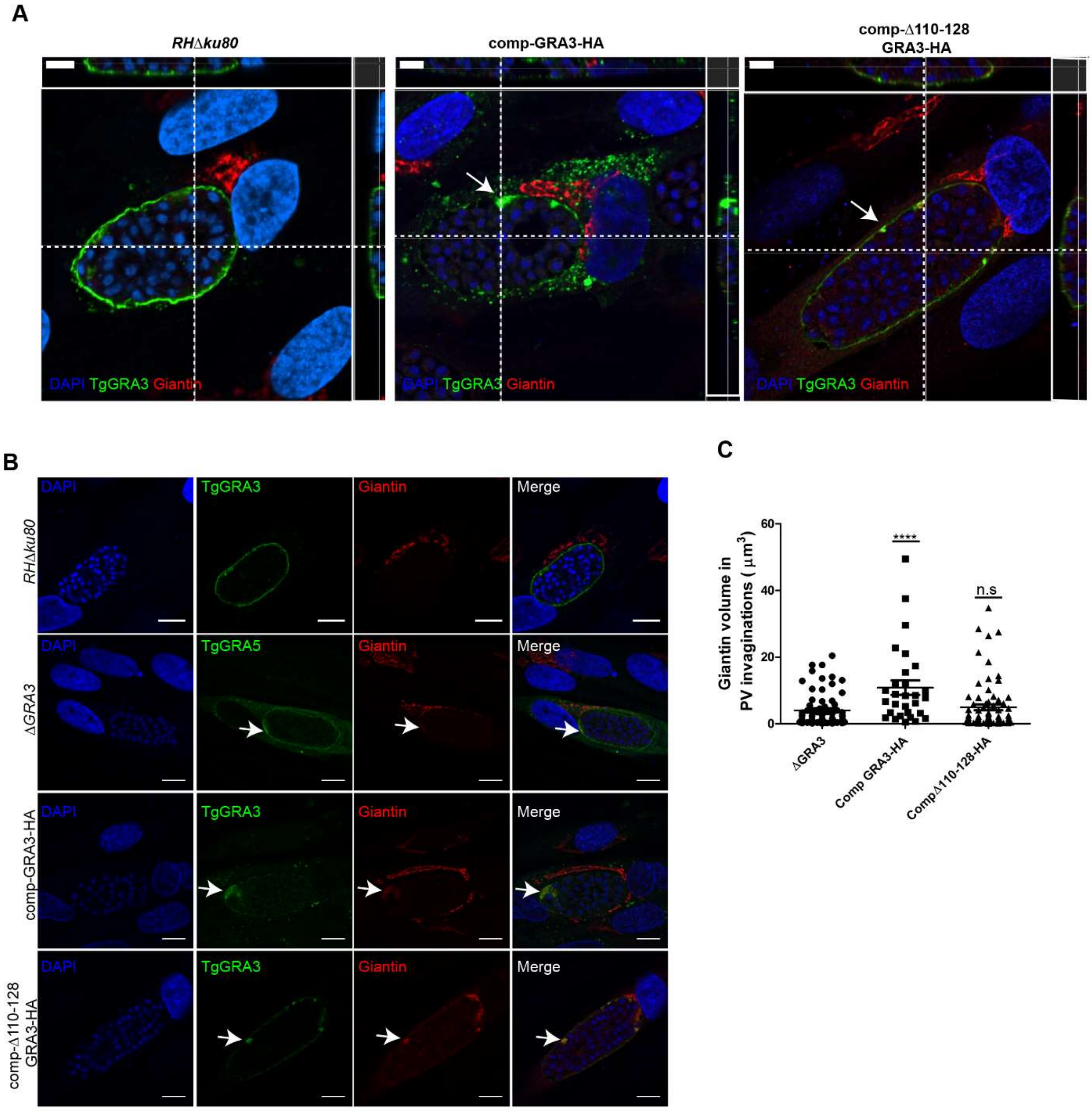

Fig. S5. TgGRA3 complementation rescued host Golgi accumulation at the PVM

(A) HFF cells were infected for 35h with *RHΔku80* parental strain, ΔGRA3 parasites strains complemented with TgGRA3 wild type sequence (comp GRA3-HA) or TgGRA3 Δ110-128 mutant sequence (comp Δ110-128-HA). Cells were fixed and stained with DAPI (nucleus, blue), antibodies specific to HA tag (green), antibodies specific to GRA3 for the parental strain (green) and giantin for the *cis*-Golgi marker (red). Orthoviews showed correct PVM localization of TgGRA3 in the three strains (white squares, upper and right panels). Dashed lines represented localization of the ortho-cuts, the horizontal lines for the right panel and the vertical line for the upper panel. White arrows indicated TgGRA3 concentrated sites at the PVM. Scale bars 5µm. (B) HFF cells were infected by *RHΔku80* parental strain, Δ*GRA3*, complemented Δ*GRA3* comp GRA3-HA, and Δ*GRA3* comp Δ110-128-HA mutants for 35h. Cells were fixed and stained with DAPI (nucleus, blue), antibodies specific to HA tag (green), TgGRA5 (green, PV marker), and giantin for the *cis*-Golgi marker (red); Scale bars 5µm. A single slice from a z-stack image was visualized. The white arrows indicated PVM invaginations containing host Golgi membranes. Scale bars, 10 µm. (C) Quantification of the volume of invaginations in the PV of Δ*GRA3,* Δ*GRA3* comp GRA3-HA, and Δ*GRA3* comp Δ110-128-HA parasite mutants. A total of 30 parasitophorous vacuoles from three independent experiments were imaged for each strain. Three-dimensional reconstruction was used to measure the invagination volume. n=72 invaginations ± s.e.m. ****$p<0.0001$ (unpaired t-test), n.s: non-significant.

Biology Open • Supplementary information

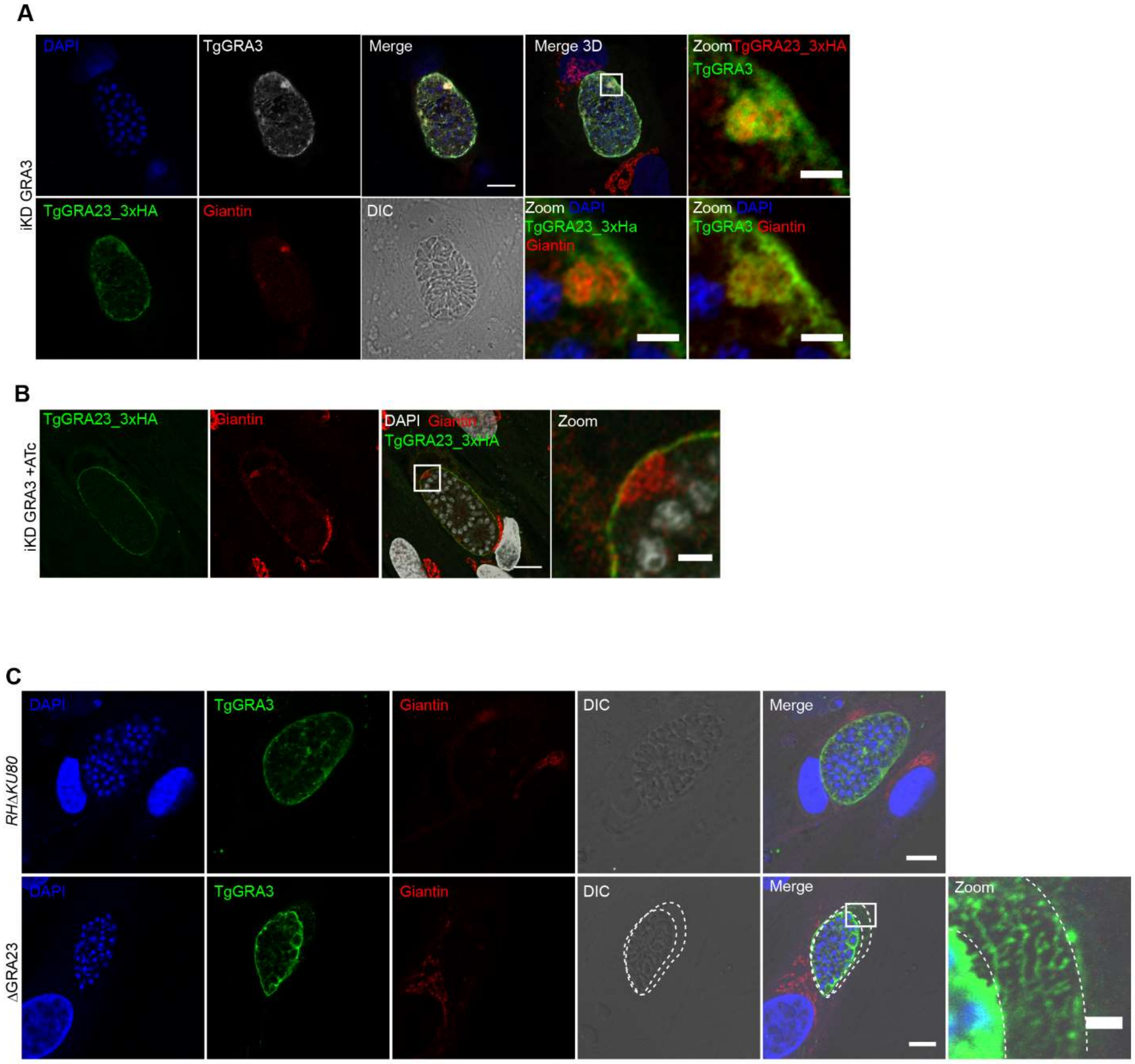

Fig. S6. TgGRA23 depletion disturbs TgGRA3 distribution at the PVM

(A) HFF cells were infected for 40h with iKD-GRA3 parasites expressing TgGRA23_3xHA. TgGRA3 (grey or green) and TgGRA23_3xHA (green or red) were localized with host Golgi accumulated material at the PVM. Cells were fixed and stained with DAPI (nucleus, blue), antibodies specific to HA tag (green), antibodies specific to TgGRA3 and giantin for the *cis*-Golgi marker (red). White square represented zoomed regions. Scale bars, 10 µm; Scale bars for zoomed regions 2 µm. (B) HFF cells were

Biology Open • Supplementary information

infected for 40h with iKD-GRA3 parasites expressing TgGRA23_3xHA in presence of ATc. Following TgGRA3 depletion, TgGRA23_3xHA did not localize with invaginated host Golgi material. Cells were fixed and stained with DAPI (nucleus, grey), antibodies specific to HA tag (green), and giantin for the *cis*-Golgi marker (red). White square represented zoomed region. Scale bars, 10 µm; Scale bars for zoomed regions 2 µm. (C) A single slice from z-stack images showing altered TgGRA3 localization in the INV of Δ*GRA2*3 mutant infected cells. HFF cells were infected with Δ*GRA23* mutant parasites. Cells were fixed and stained with DAPI (nuclei, blue) and antibodies specific to giantin for the cis-Golgi marker (red), or anti-GRA3 (green). The abnormal intravacuolar space was observed in the DIC image. White dashed circles delineate the PVM and the parasite rosette. White square represented zoomed region. Scale bars, 10µm. Scale bars for zoomed regions 2 µm.

Biology Open • Supplementary information

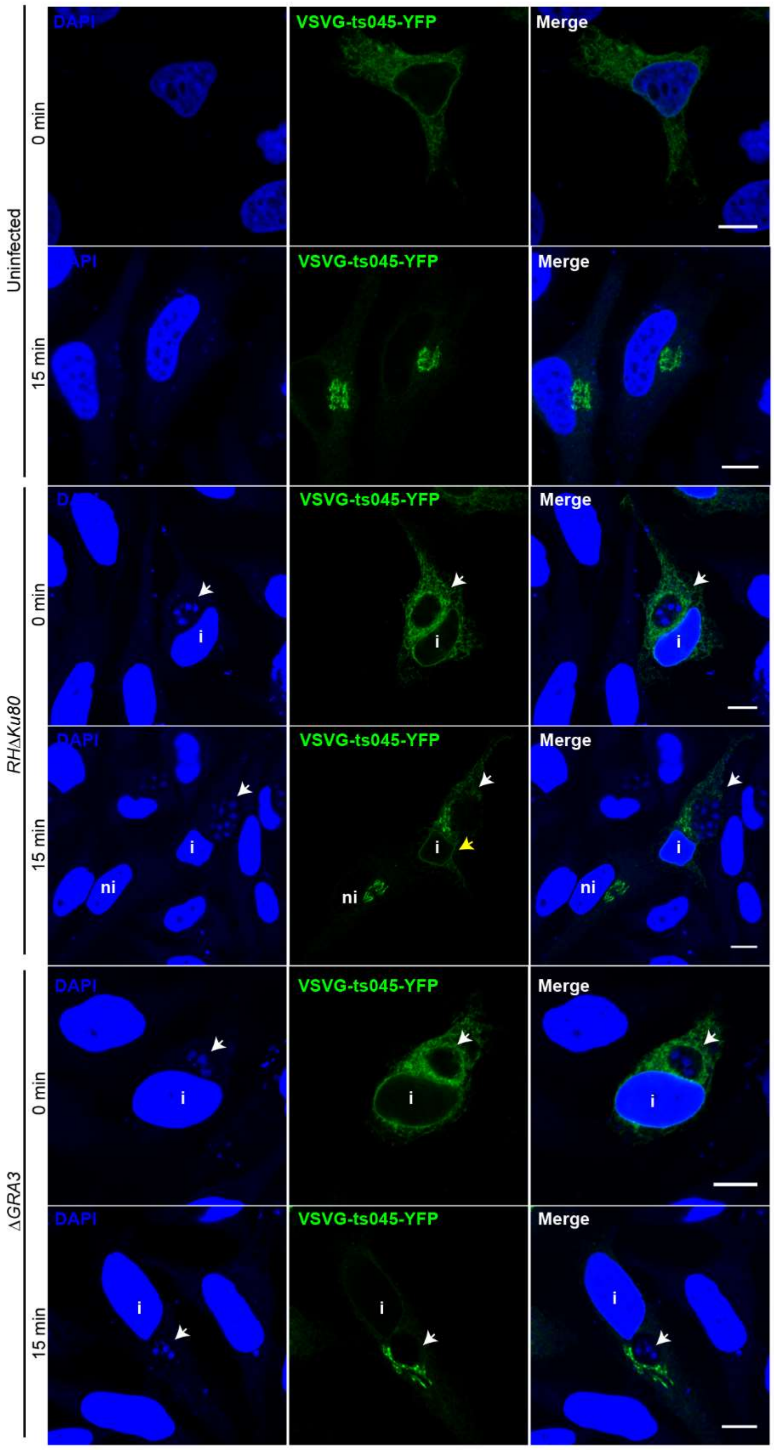

Fig. S7. TgGRA3 disturb trafficking from ER to Golgi

HFF cells were infected for 30 h with *RHΔKu80* parental strain and Δ*GRA3* parasites. Representative z-stack confocal images showed VSVG-ts045-YFP (green) at the ER at 39°C, then after 15 min at permissive temperature (32°C), it was localized at the host Golgi. White arrows showed the parasitophorous vacuole, the yellow arrow indicated VSVG-ts045-YFP at the ER and the host Golgi after 15min. ni, non-infected cell; i, infected cell. Brightness of DAPI staining was increased to visualize parasites nuclei. Scale bars, 10μm.

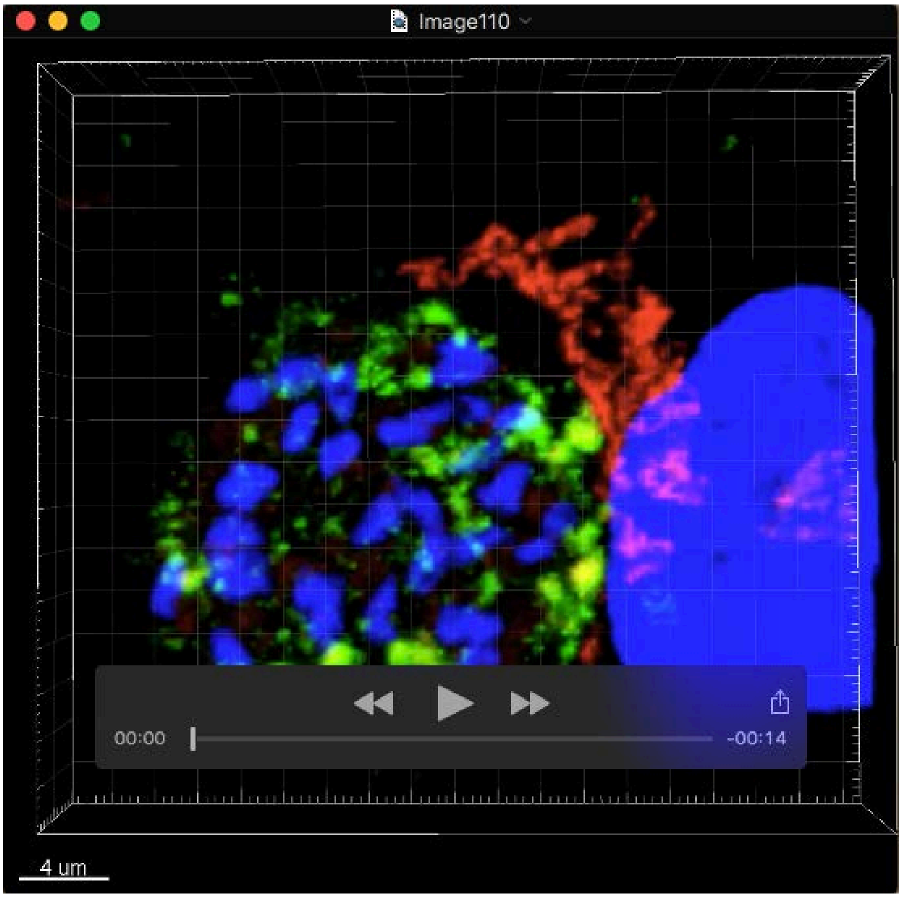

Movie 1. TgGRA3 localizes in tubules inside the PV containing host Golgi material

3D reconstruction from Fig 2C. Z-stacks images carried out by confocal microcopy were used for 3D reconstruction. It indicated that TgGRA3 (green) coated tubules containing host Golgi material inside the PV (GCC185, red).

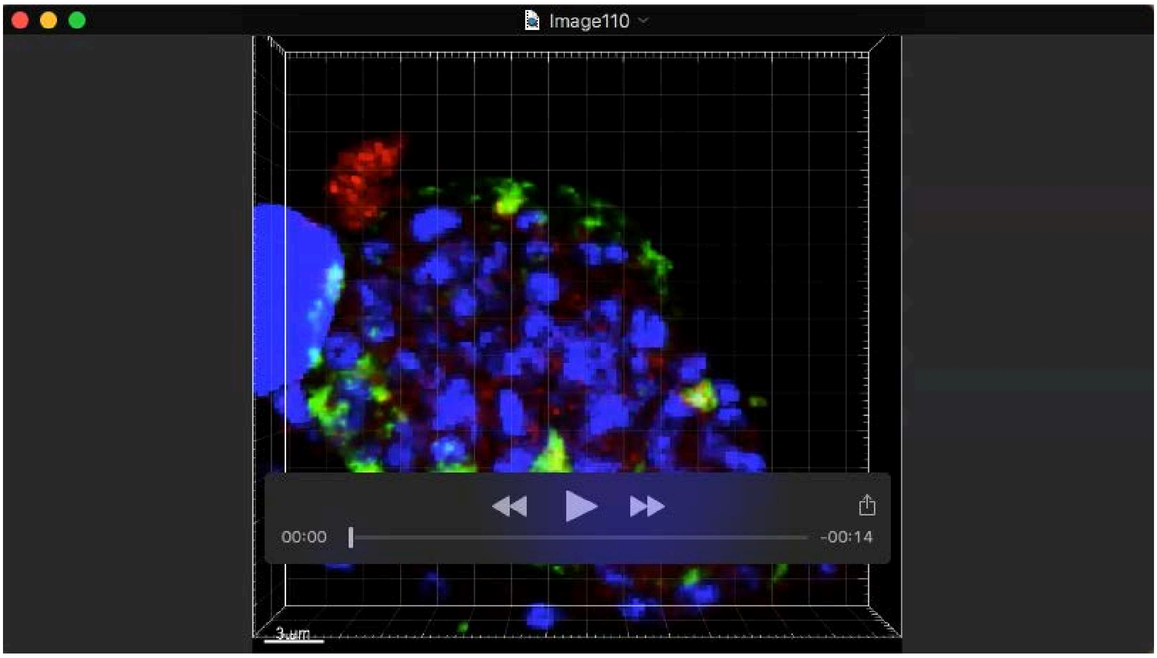

Movie 2. TgGRA3 localizes in structures inside the PV containing host Golgi material

3D reconstruction from another vacuole showing TgGRA3 (green) coated structures containing host Golgi material inside the PV (GCC185, red).

Biology Open • Supplementary information

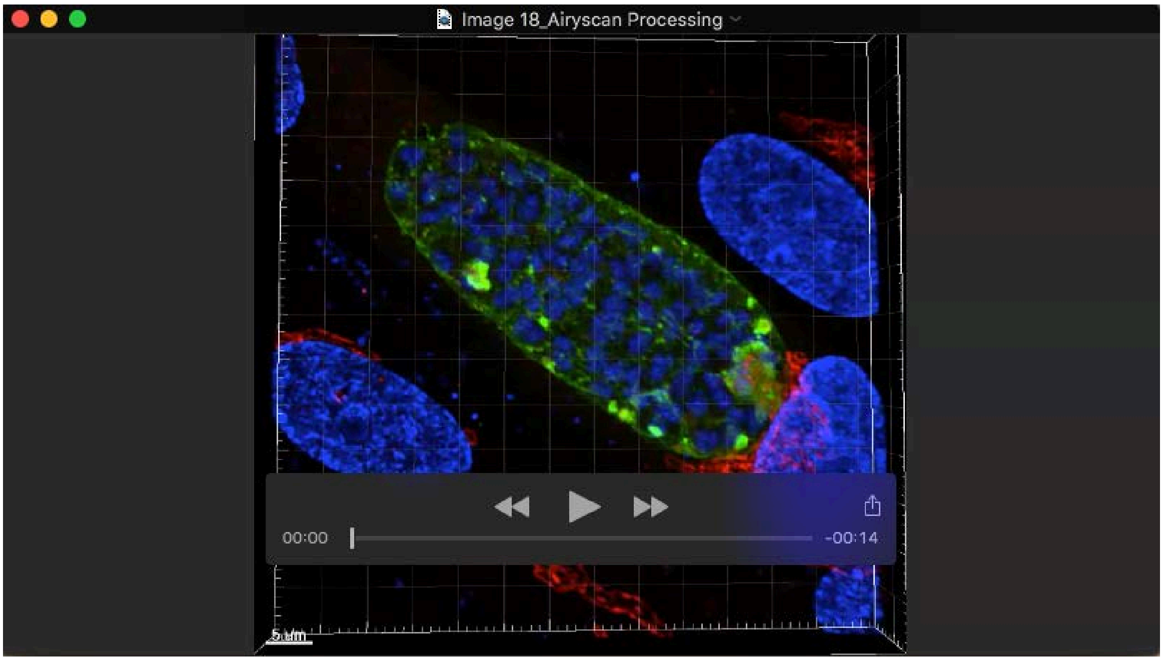

Movie 3. GRA3 comp Δ110-128 mutant did not rescue the host Golgi accumulation in the Δ*GRA3*

3D reconstruction from Fig S5B was carried out with z-stacks obtained by confocal microscopy. GRA3 comp Δ110-128_HA was labeled with anti-HA antibody (green), the host Golgi (Giantin, red) and nuclei (DAPI, blue).

Table S1. Comparative list of *Toxoplasma gondii* or *Cricetulus griseus* proteins identified per sample (Control, Golgi or Reticulum) by nanoLC-MS/MS Orbitrap mass spectrometer (Q-Exactive)

Click here to Download Table S1

Table S2. List of selected parasite proteins that bind to host Golgi

Click here to Download Table S2

Table S3. List of oligonucleotides used during this work

The name and sequence of the oligonucleotides.

Name	Primers
PDTS4KOGRA3_3UTR_F	5'AATAGATCTATGTACCCATACGATGTTCCAGATTACGCT3'
PDTS4KOGRA3_3UTR_R	5'TCAACTCCTAGGCTTCAACGTTC3'
PDTS4KOGRA3_5UTR_F	5'CGCCATATGACCATGATAATTGTACAGCT3'
PDTS4KOGRA3_5UTR_R	5'AATCATATGGAATCTTCTCCGCTCCGCTGCGTCG3'
pLicGRA23_3xHA_F	5'TACTTCCAATCCAATTTAATGCCTCGTAGCACCAGCGTCGTAG T3'

Biology Open • Supplementary information

pLicGRA23_3xHA_R	5'TCCTCCACTTCCAATTTTAGCGTTCTTTCGCGCAAGGGGTTTCTT3'
pLicROP13_3xHA_F	5'TACTTCCAATCCAATTTAATGCGTCACTGTCTTTCACTGAAGGC3'
pLicROP13_3xHA_R	5'TCCTCCACTTCCAATTTTAGCCAATAGCCTCAAGGAATTCGCATAG3'
pGEX6-P3_GRA3rec_F	5'CCTGGGATCCATGGACCGTACC3'
pGEX6-P3_GRA3rec_R	5'CGGGGATCCTCAGGTTTGTTTCTTG3'
KOGRA23_5UTR_F	5'CTGGGTACCTGTGCGAAGCCGCA3'
KOGRA23_5UTR_R	5'CCCATCGATTGGTGGATTTGGTGTATCT3'
KOGRA23_3UTR_F	5'GGGGGATCCGACGAAGGGTCCGAC3'
KOGRA23_3UTR_R	5'GTGGCGGCCGCTTTTGAGAATTGCCG3'
KOGRA3_5UTR_F	5'GGGCCCACCATGATAAATTGTACAGCTTGT3'
KOGRA3_5UTR_R	5'AAGCTTTTGTTACTCAGCGCAAGTGA3'
KOGRA3_3UTR_F	5'ACTAGTAAACATTCGTTGACGCAGAT3'
KOGRA3_3UTR_R	5'GCGGCCGCACACACTGGTCTTGTTCGAT3'
ROP13_F (1)	5'GGATCCATGAAGAGAACAGAGCTTTGTATCGCA3'
ROP13_R (2)	5'GCGGCCGCTCACAATAGCCTCAAGGAATTCGCA3'
ROP13gen_R (4)	5'GACAGAAGAGCGACTCGGTGATTG3'
GRA23gen_F (1)	5'GAAAAGAAAGCGGTGAGGGCCGTC3'
GRA23_R (2)	5'GATGCGGCCGCCTAGTTCTTTCGCGCAAGGGG3'
GRA23_F (3)	5'CTGGGATCCATGGCAGCGCGTGCG3'
GRA23gen_R(4)	5'GCGGTTCCTCACATGAAAAATGCAACG3'
HXGPRT_R up (5)	5'GGGCGGGTTTGAATGCAAGGTTT3'
HXGPRT_F down (6)(3)	5'CTCCCTTAACTAAATAAGCCGCGACACC3'
GRA3gen_F (1)	5'AAGGCCAAATGAAGCATTTACAGGCCT3'
DHFR_R (2)	5'GGGCGGGTTGAATGCAAGGTTT3'

Biology Open • Supplementary information

GRA3_F (3)	5'CCTGGGATCCATGGACCGTACC3'
GRA3_R (4)	5'CGGGGATCCTCAGGTTTGTTTCTTG3'
GRA1_prom_F	5'GGCGCGCCATGGACCGT3'
GRA1_prom_R	5'GGCGCGCCACGCACCATCTT3'
HA-GRA3_F	5'GGATCCTACCCATACGATGTTCCAGATTACGCTGATCAGCCTG AAAATCATCAGGCT3'
∆43-71_F	5'GAGTCATACAGTTCTGCAACTTCGGGTGTCCAAGAAGC3'
∆43-71_ R	5'GCGGCCGCTCATATATTGTCTCGCAACGTCC3
∆126-143_F	5'TCTCTTGCGACAAGTGCAGCGATAGGT3'
∆126-143_R	5'TTCCCGCCTCAATAACGACAACTCCTC3'
∆110-143_ F	5'GATCGCACAGATCGCCCTGG3'
∆110-143_ R	5'ACCTCCCTCCGCCTGATTGCCA3'
comp_GRA3_F	5'GGCGCGCCATGGACCGT3'
comp_GRA3_R	5'CCCGGGGGTTTGTTTCTTGGAGG3'

Biology Open • Supplementary information
